# Mucin expression in pancreatic ductal adenocarcinoma cell lines in 2D and 3D cultures: A proteomic and immunocytochemical analysis

**DOI:** 10.1371/journal.pone.0353991

**Published:** 2026-07-16

**Authors:** Yuuki Shichi, Hiroki Tsumoto, Masakazu Fujiwara, Keisuke Nonaka, Yasuko Hasegawa, Seiichi Shinji, Hirofumi Rokutan, Kimimasa Takahashi, Tomio Arai, Yuri Miura, Toshiyuki Ishiwata

**Affiliations:** 1 Division of Aging and Carcinogenesis, Research Team for Geriatric Pathology, Tokyo Metropolitan Institute for Geriatrics and Gerontology, Tokyo, Japan; 2 Research Team for Mechanism of Aging, Tokyo Metropolitan Institute for Geriatrics and Gerontology, Tokyo, Japan; 3 Department of Gastroenterological Surgery, Nippon Medical School, Tokyo, Japan; 4 Department of Pathology, Tokyo Metropolitan Institute for Geriatrics and Gerontology, Tokyo, Japan; 5 Department of Veterinary Pathology, School of Veterinary Medicine, Nippon Veterinary and Life Science University, Tokyo, Japan; University of Health Sciences Lahore, PAKISTAN

## Abstract

Pancreatic ductal adenocarcinoma (PDAC) exhibits diverse phenotypes, including epithelial and mesenchymal characteristics, yet these features have not been effectively translated into clinical applications. Mucins are implicated in tumor progression and therapeutic resistance and are considered potential diagnostic and therapeutic targets. In this study, five epithelial and three mesenchymal PDAC cell lines were cultured under two-dimensional (2D) and three-dimensional (3D) conditions to investigate mucin expression. Proteomic analysis identified five mucins (MUC1, MUC4, MUC5B, MUC19, and MUC20) in 2D culture and eight (including MUC2, MUC5AC, and MUC13) in 3D culture. Candidate mucins were further validated by immunocytochemistry with H-score assessment. MUC1 was consistently expressed in all PDAC cell lines and showed marked upregulation in several lines under 3D culture. In mesenchymal PDAC cell lines, mucin expression was largely restricted to MUC1, whereas epithelial lines displayed broad 3D-induced reorganization. Notably, MUC5AC was absent in 2D culture but robustly induced in all epithelial PDAC cell lines under 3D conditions. Other mucins, including MUC2, MUC4, MUC5B, MUC13, MUC19, and MUC20, were variably upregulated, with epithelial lines demonstrating higher diversity and intensity of expression. These findings demonstrate that 3D culture effectively reveals the plasticity and heterogeneity of mucin expression in PDAC, highlighting its potential as a platform for biomarker discovery and the development of therapeutic strategies.

## Introduction

Pancreatic ductal adenocarcinoma (PDAC) remains difficult to detect at an early stage, and surgery is the only curative option; nevertheless, long-term outcomes are poor, with a 5-year survival of ~12% and frequent postoperative recurrence [1]. Most patients present with unresectable disease, and marked inter- and intra-tumoral heterogeneity further complicates biomarker development and therapy selection [2,3].

Mucins are heavily glycosylated high-molecular-weight glycoproteins that constitute the mucus barrier and modulate epithelial adhesion, polarity, signaling, and immune interactions [4]. Among the mucins evaluated here—membrane-bound (MUC1, MUC4, MUC13) and secreted (MUC2, MUC5AC, MUC5B, MUC19, MUC20)—MUC1 has been linked to invasion and metastasis, MUC4 can alter ErbB/EGFR signaling and drug responses, and MUC13 promotes growth and migration; secreted mucins such as MUC2, MUC5AC, MUC5B, MUC19, and MUC20 contribute to gel formation and the viscoelastic properties of secretions [5–9]. Although individual mucins have been described in PDAC tissues and cell lines, systematic, protein-level comparisons across multiple PDAC lines under matched two-dimensional (2D) and three-dimensional (3D) conditions remain limited, particularly with regard to quantitative scoring and subcellular localization.

PDAC cells exist along epithelial-like (E-cadherin–high) and mesenchymal-like (vimentin–high) states that associate with distinct phenotypes [10,11]. 3D culture recapitulates tissue architecture better than 2D by restoring cell–cell/cell–matrix interactions and microenvironmental gradients and can reprogram transcriptional and proteomic programs. Indeed, epithelial and mesenchymal PDAC cell lines form distinct spheres and display differential drug responses in 3D [12,13]. We previously noted that MUC1 and MUC5AC transcripts increase in 3D relative to 2D in the epithelial PDAC cell line PK-8, suggesting context-dependent plasticity of mucin expression [14].

Mucins may be particularly susceptible to remodeling during the transition from 2D to 3D culture. This is because mucin biosynthesis depends on epithelial polarity, Golgi-resident mucin-type O-glycosylation, and polarized secretory trafficking [15–17]. These epithelial features are not fully recapitulated in flat monolayer cultures, whereas 3D spheres can partially restore tissue-like architecture, cell–cell interactions, and apical–basal polarity, while also generating gradients of oxygen and nutrients [18–20]. In addition, hypoxic and stress-responsive microenvironments in 3D cultures may contribute to the up-regulation of selected membrane-tethered and gel-forming mucins through HIF-1α-dependent pathways and endoplasmic reticulum stress or unfolded protein response signaling [21–24]. We therefore hypothesized that 3D culture differentially remodels mucin expression programs in epithelial versus mesenchymal PDAC cell lines. In this study, both proteomic and immunocytochemical analyses were designed to assess mucin protein cores rather than glycan structures. To test this, we profiled eight PDAC cell lines. First, we used proteomics to identify the mucins expressed in these models. We then validated and semi-quantitatively assessed protein expression levels and subcellular localization of the identified mucins (MUC1, MUC2, MUC4, MUC5AC, MUC5B, MUC13, MUC19, MUC20) by immunocytochemistry on cell-block preparations under 2D monolayer and 3D sphere conditions, using H-scores (0–300) for comparative evaluation. This approach delineates line-specific and state-dependent mucin programs in PDAC and establishes a framework for interpreting mucin biology in in-vitro model systems.

## Materials and methods

### Reagents

RapiGest SF was purchased from Waters (Milford, MA, USA). Triethylammonium bicarbonate (TEAB) buffer, dithiothreitol, iodoacetamide, Pierce 660 nm Protein Assay Reagent, and Ionic Detergent Compatibility Reagent were purchased from Thermo Fisher Scientific (Waltham, MA, USA). GL-Tip SDB was purchased from GL Sciences (Tokyo, Japan). Ultrapure water, acetonitrile (MeCN), and formic acid (FA) for liquid chromatography-mass spectrometry were purchased from FUJIFILM Wako Pure Chemical Corporation (Osaka, Japan). Trypsin platinum, mass spectrometry grade, was purchased from Promega (Madison, WI, USA).

### Experimental design

**Fig 1:**
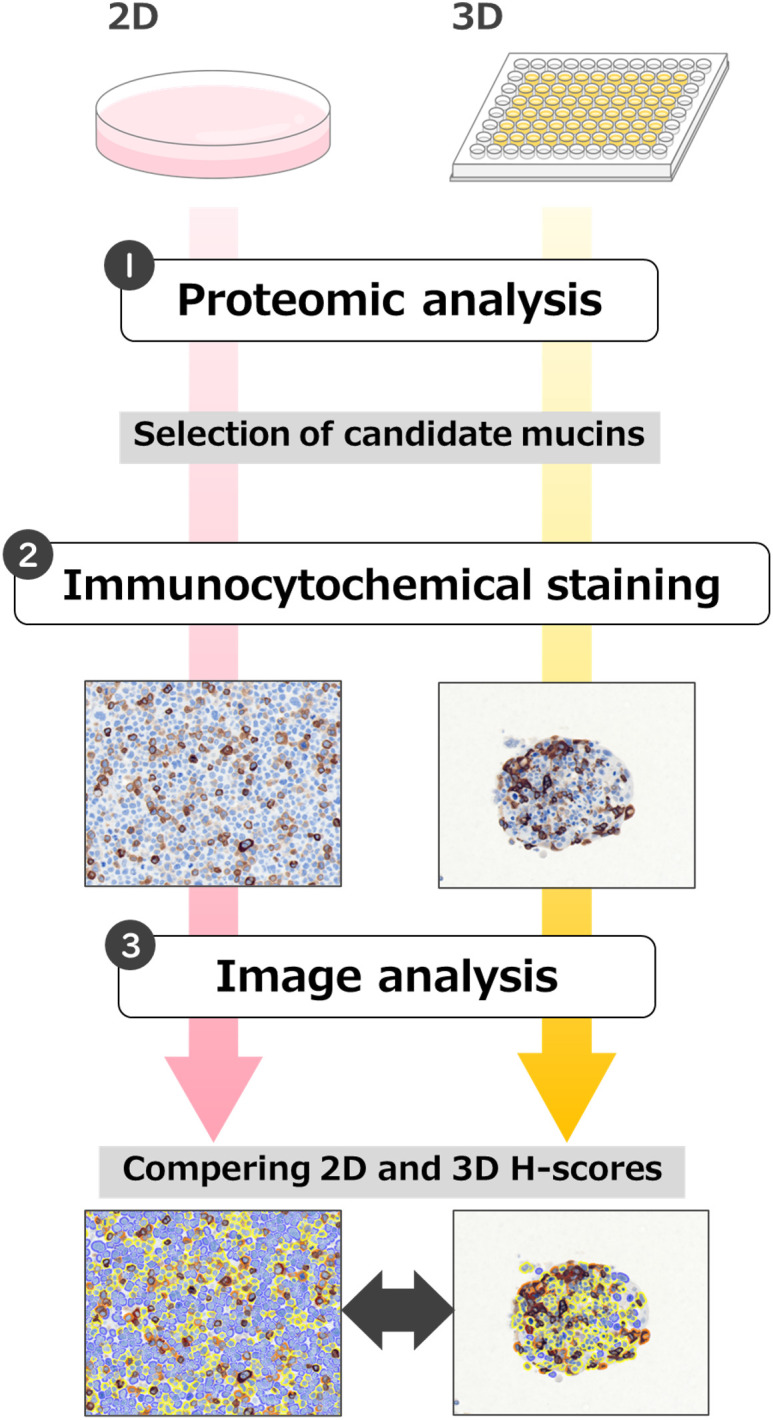
Schematic overview of the experimental design. Mucin expression profiles were identified by the proteomic analysis of eight human pancreatic ductal adenocarcinoma (PDAC) cell lines cultured under 2D and 3D conditions. Candidate mucins were validated by immunocytochemical staining using specific antibodies on cell blocks prepared from each cell line. The H-score was calculated by integrating both the proportion of positively stained cells and the intensity of immunoreactivity.

Proteomic analysis was first performed on eight PDAC cell lines cultured in 2D and 3D to screen for candidate mucins that were expressed (Fig 1). Subsequently, we used PDAC cell blocks from 2D- and 3D-cultured cell lines for immunocytochemical staining using specific antibodies against the candidate mucins. Image analysis of the immunostaining results identified mucins produced by PDAC cells. We then compared the types of mucins produced by PDAC cells in 2D and 3D cultures.

### Cell culture

Human epithelial PDAC (PK-8, PK-45P, and T3M-4) and mesenchymal PDAC (KP4) cell lines were provided by the RIKEN BRC through the National Bio-Resource Project of the MEXT/AMED, Japan. Human epithelial PDAC (PK-59 and PK-1) and mesenchymal PDAC (PANC-1 and MIA PaCa-2) cell lines were obtained from the Cell Resource Center for Biomedical Research, Institute of Development, Aging and Cancer, Tohoku University (Sendai, Japan). These eight cell lines were selected to include both epithelial-classical and mesenchymal-quasi-mesenchymal phenotypes, which are commonly distinguished in transcriptomic classifications of PDAC cell lines. The cells were cultured in a growth medium (RPMI-1640 medium supplemented with 10% fetal bovine serum) at 37°C in a humidified 5% CO_2_ atmosphere. For 2D cultures, adherent cells were collected by trypsinization, centrifuged (1500 rpm, 5 min), and washed with phosphate-buffered saline. This procedure was performed twice. For 3D cultures, PDAC cells were seeded at 3.0 × 10^3^ cells/well in low-attachment 96-well plates (Thermo Fisher Scientific) for seven days. Detailed protocols for 3D sphere formation are available elsewhere [25,26]. Both 2D and 3D cultures of PDAC cells used the same culture medium but were grown in different culture plates. The growth medium was not exchanged during the seven-day 3D culture period. Sphere morphology at day 7 varied among the cell lines. The epithelial PDAC cell lines (PK-1, PK-8, PK-45P, PK-59, and T3M-4) formed compact, well-circumscribed spheres, whereas KP4 and PANC-1 formed grape-like aggregates with discernible cell–cell contacts. MIA PaCa-2 cells formed loose aggregates rather than compact spheres. Using the *Mycoplasma* PCR Detection Kit (iNtRON Biotechnology, Jungwon-Gu, South Korea), we confirmed that none of the cells had *Mycoplasma* contamination. Genomic DNA was extracted from PDAC cells using a DNeasy Blood and Tissue Kit (Qiagen, Hilden, Germany) following the manufacturer’s protocol. Short tandem repeats were analyzed using the GenePrint 10 System (Promega, Madison, WI, USA), following the manufacturer’s protocol. All PDAC cell lines were correctly genotyped and demonstrated no contamination.

### Proteomic analysis

Sample preparation for proteomic analysis was conducted twice for eight PDAC cell lines, and each sample underwent liquid chromatography-tandem mass spectrometry (LC-MS/MS) analysis thrice.

Cell pellets from 2D and 3D cultures were suspended in 100 μL of 50 mM TEAB buffer (pH 8.5) containing 0.5% RapiGest SF (lysis buffer), sonicated, incubated at 95°C for 5 min, and centrifuged (16,000 × g, 20 min, 23°C). The supernatants were collected, and protein concentrations were measured using Pierce 660 nm Protein Assay Reagent with Ionic Detergent Compatibility Reagent. The supernatants containing 20 μg of protein were diluted with lysis buffer up to 51 μL. Subsequently, 3 μL of 100 mM dithiothreitol was added, and the mixture was incubated at 95°C for 5 min. After incubation, 6 μL of 100 mM iodoacetamide was added, and the mixture was incubated at 23°C for 20 min in the dark. The reduced and alkylated sample was diluted with 195 μL of 50 mM TEAB buffer (pH 8.7). Subsequently, 4 μL of 0.5 μg/μL trypsin (protein/trypsin, 10:1) was added, and the mixture was incubated at 37°C for 18 h. Trypsin digestion was performed using Trypsin Platinum, Mass Spectrometry Grade (#VA9000; Promega), at a protein-to-trypsin mass ratio of 10:1 according to the manufacturer’s protocol. The digested sample was acidified with 12 μL of 10% trifluoroacetic acid, incubated at 37°C for 30 min, centrifuged (13,000 rpm, 10 min, 23°C), and desalted using GL-Tip SDB. The eluates were evaporated *in vacuo* and resuspended in 80 μL of 5% MeCN containing 0.1% FA at a concentration of 0.25 μg/μL.

LC-MS/MS analysis of tryptic peptides was performed using an Ultimate 3000 RSLCnano system coupled to a Q Exactive mass spectrometer (Thermo Fisher Scientific). PepMap Neo (0.3 × 5 mm, 5 μm particle size, C18; Thermo Fisher Scientific) and NANO HPLC Capillary Column (75 μm × 12 cm, 3 μm particle size, C18; Nikkyo Technos, Tokyo, Japan) were used as the trap and analytical columns, respectively. Peptide separation was performed using water containing 0.1% FA (solvent A) and MeCN containing 0.1% FA (solvent B) at a flow rate of 300 nL/min. The linear gradient for peptide separation was as follows: 0–3 min, 2% B; 3–123 min, 2–40% B; 123–125 min, 40–95% B; 125–135 min, 95% B; 135–137 min, 95–2% B; and 137–150 min, 2% B. A 2 μL sample (0.5 μg protein) was injected. The mass spectrometer was operated in data-dependent acquisition (DDA) mode. All mass spectra were acquired with the following settings: spray voltage, 2.0 kV; capillary temperature, 275°C; S-lens radio frequency level, 50; polarity, positive ion; resolution, 70,000; automatic gain control (AGC) target, 3e6; maximum ion injection time (IT), 100 ms; and scan range, *m*/*z* 350–1,500. The DDA parameters were as follows: resolution, 17,500; AGC target, 1e5; maximum IT, 100 ms; loop count, 10; isolation window, 1.6 *m*/*z*; normalized collision energy, 27; dynamic exclusion, 15 s; and charge exclusion, unassigned, 1, 8, and > 8.

LC-MS/MS data were analyzed using the Proteome Discoverer 2.4 software (Thermo Fisher Scientific). Peptide-spectrum matches, peptides, and proteins were filtered using Percolator, Peptide Validator, and Protein FDR Validator, respectively, with strict and relaxed false discovery rate (FDR) thresholds of 0.01 and 0.05. The parameters for protein identification were as follows: search engine, Sequest HT; parent mass tolerance, 10.0 ppm; fragment mass tolerance, 0.02 Da; enzyme, trypsin (full); maximum missed cleavage sites, 2; static modification, carbamidomethyl (Cys, + 57.021 Da); dynamic modification, oxidation (Met, + 15.995 Da); and protein database, Homo sapiens (SwissProt, reviewed, 20,379 sequences). Parameters for label-free quantification (LFQ) were as follows: precursor abundance based on, area; normalization mode, total peptide amount; protein ratio calculation, pairwise ratio based; hypothesis test, t-test (background based). Total-peptide-amount normalization was used to correct for differences in overall peptide abundance among LC-MS/MS runs.

### Immunocytochemical analysis

Adherent PDAC cells were collected after trypsin treatment, centrifuged (1,500 rpm, 5 min), and fixed with 10% neutral-buffered formalin for 3 h. For sphere formation in 3D culture, PDAC cells were seeded in a growth medium at 3.0 × 10^3^ cells/well in 96-well low-attachment plates (174925; Thermo Fisher Scientific). After seven days, the spheres were aspirated using micropipettes and fixed in formalin for 3 h. Formalin was removed using a micropipette, and both PDAC cells and spheres were dehydrated in graded ethanol and embedded in paraffin. Subsequently, serial sections of the cell blocks (3-μm thick) were stained using the Histofine Simple Stain MAX-PO (Multi) Kit (Nichirei Biosciences, Tokyo, Japan). For MUC1, MUC2, MUC4, MUC5AC, MUC13, MUC19, and MUC20, antigen retrieval was performed using heat treatment with the retrieved antigen solution (Nichirei Biosciences). The primary antibodies used for immunocytochemical staining were as follows: mouse monoclonal anti-MUC1 (Ma695; Sanbio B. V., Uden, The Netherlands; Cat# MONX10514; 1:100), mouse monoclonal anti-MUC2 (Ccp58; Leica Biosystems, Wetzlar, Germany; Cat# PA0155; ready-to-use), mouse monoclonal anti-MUC4 (8G7; Santa Cruz Biotechnology, Texas, CA, USA; Cat# sc-53945; 1:400), mouse monoclonal anti-MUC5AC (CLH2; Leica Biosystems; Cat# NCL-MUC-5 AC; 1:100), mouse monoclonal anti-MUC5B (C10; Invitrogen, Waltham, MA, USA; Cat# MA5–41641; 1:100), mouse monoclonal anti-MUC13 (D-5; Santa Cruz; Cat# sc-373857; 1:200), mouse monoclonal anti-MUC19 (876013; R&D Systems, Minneapolis, MN, USA; Cat# MAB8245; 1:400), and rabbit polyclonal anti-MUC20 antibody (Invitrogen; Cat# PA5–98640; 1:500). Antigen retrieval was performed by heat treatment at 98˚C for 40 min using BOND Epitope Retrieval Solution 1 (AR9961; Leica Biosystems) for MUC1, MUC5AC, MUC19, and MUC20, and BOND Epitope Retrieval Solution 2 (AR9640; Leica Biosystems) for MUC2, MUC4, and MUC13. No antigen retrieval was performed for MUC5B. Endogenous peroxidase activity was blocked by treatment with 0.3% H2O2 in water at 23˚C for 5 min. The sections were then incubated with each primary antibody for 15 min at 23˚C. Antigen detection was performed using 3,3′-diaminobenzidine tetrahydrochloride, followed by counterstaining with hematoxylin. Negative controls were generated by omitting the primary antibodies.

Images were captured using a Matra multispectral microscope (PhenoImager Mantra; AKOYA Biosciences, Marlborough, MA, USA) and analyzed using the dedicated software inForm 2.4 (AKOYA Biosciences). The staining intensity was classified into four ordered categories: 0 (poor), 1+ (weak), 2+ (moderate), and 3+ (strong). The H-score (0–300) for each specimen was defined as (1 × percentage of 1 + cells) + (2 × percentage of 2 + cells) + (3 × percentage of 3 + cells); cells scored 0 contribute 0 to the calculation (S1 Fig). Percentages were expressed on a 0–100% scale. For interpretability, H-scores were further stratified into four levels: 0–5 (negative), > 5–50 (weakly positive), > 50–150 (positive), and >150–300 (strongly positive). For 2D culture, 2,000–3,000 cells were evaluated across four non-overlapping fields of view per sample. For 3D culture, ten spheres (each comprising approximately 100–1,000 cells) were analyzed for every cell line.

### Statistical analysis

Comparisons of H-scores between 2D- and 3D-cultured conditions were performed for each mucin type in each PDAC cell line. Statistical significance was evaluated using unpaired t-tests, Welch’s t-tests, or Mann–Whitney U tests, depending on the distribution and variance of the data. A two-tailed *P* value < 0.05 was considered statistically significant in the primary analysis. Raw *P* values were further adjusted across 64 cell-line × mucin comparisons using the Benjamini–Hochberg false-discovery-rate procedure, and adjusted q values < 0.05 were considered statistically significant after correction. The statistical test used for each comparison, mean H-scores, raw *P* values, adjusted q values, and significance after correction are provided in S1 Table.

An H-score >5 was used as the threshold for positivity in the present study because no universally accepted H-score cutoff has been established for these mucin antibodies in pancreatic cancer cell lines. This value is approximately equivalent to weak staining in 5% of cells and is consistent with previous mucin immunohistochemical studies using 5% positivity as a cutoff. All statistical analyses were conducted using GraphPad Prism software (version 10.6.1 [892]; GraphPad Software, San Diego, CA, USA) and Microsoft Excel. *P < 0.05, **P < 0.01, ***P < 0.001.

## Results

### Proteomic analysis of mucin expression in 2D- and 3D-cultured PDAC cell lines

To detect mucins expressed under different culture conditions, we performed proteomic analyses using eight PDAC cell lines in two independent experiments (Table 1). In 2D-cultured cells, five mucins (MUC1, MUC4, MUC5B, MUC19, and MUC20) were detected, whereas in 3D-cultured cells a total of eight mucins—including MUC2, MUC5AC, and MUC13—were detected. Both the total number of peptides and the number of unique peptides were generally higher in 3D than in 2D culture. Two shared peptides were observed among the eight mucins: one shared between MUC5AC and MUC5B, and another shared among MUC2, MUC5AC, and MUC5B. To assess differential expression, mass spectrometry-based LFQ was performed and the protein abundance ratio (3D/2D) of eight mucins were calculated (Table 2). To evaluate the effect of total-peptide-amount normalization on mucin quantification, normalized and non-normalized mucin abundance values were compared. Although normalization increased the absolute abundance values of seven mucins, excluding MUC13, by an average factor of 1.9 ± 0.3, the corresponding 3D/2D ratios were largely preserved, with an average normalized/non-normalized ratio of 1.1 ± 0.2. In many cell lines, 3D culture was associated with an increase in mucin expression.

**Table 1:** Summary of mucins detected through proteomic analysis in 2D- or 3D-cultured PDAC cell lines.

Accession number	Proteins	Type	Number of amino acids	Molecular weight (kDa)	Sequence coverage (%)		Number of peptides		Number of unique peptides	
					2D	3D	2D	3D	2D	3D
P15941	MUC1	Membrane-bound	1255	122.0	5.3	6.1	5	6	5	6
Q02817	MUC2	Secreted	5179	540.0	−	0.2	−	1	−	0
Q99102	MUC4	Membrane-bound	2169	231.4	6.6	9.3	8	11	8	11
P98088	MUC5AC	Secreted	5654	585.2	−	9.9	−	21	−	19
Q9HC84	MUC5B	Secreted	5762	596.0	0.3	4.0	1	8	1	6
Q9H3R2	MUC13	Membrane-bound	512	54.6	−	5.9	−	2	−	2
Q7Z5P9	MUC19	Secreted	8384	804.8	0.2	0.2	1	1	1	1
Q8N307	MUC20	Membrane-bound	709	71.9	24.1	28.1	1	3	1	3

**Table 2:** Abundance ratio (3D/2D) of mucins identified by proteomic analysis in PDAC cell lines. Red and blue shading indicate upregulation and downregulation in 3D culture, respectively. “100” indicates that the protein was detected only under 3D culture, whereas “0.01” indicates that the protein was detected only under 2D culture. Dashes (—) indicate undetected proteins. Asterisks denote statistical significance: *P < 0.05, **P < 0.01, and ***P < 0.001.

		Ratio (3D/2D)															
		MUC1		MUC2		MUC4		MUC5AC		MUC5B		MUC13		MUC19		MUC20	
**Epithelial**	**PK-8**	5.22	***	2.08		0.30		1.16		1.69		−		0.82		5.18	**
	**PK-45P**	3.40	***	9.88	***	0.49		2.97	**	3.03	**	−		0.95		100	***
	**PK-59**	2.33	**	5.08	***	1.60		3.04	***	1.59		2.06	*	0.94		0.78	
	**PK-1**	1.28		1.37		0.37	**	1.25		1.06		0.94		0.80		2.27	*
	**T3M-4**	0.94		0.01	***	100	***	1.54		1.34		1.63		0.80		6.18	***
**Mesenchymal**	**PANC-1**	1.53	*	100	***	1.03		1.05		0.38	**	2.03	*	0.85		−	
	**KP4**	1.15		0.75		2.11		1.36		1.44		0.66		1.10		2.35	
	**MIA PaCa-2**	1.17		0.01	***	100	***	0.88		1.17		1.37		0.94		100	***

### Immunocytochemical analysis of 2D- and 3D-cultured PDAC cell lines

The protein expression levels and subcellular localization of eight mucins identified by proteomic analyses were further evaluated by immunocytochemistry using specific antibodies (Table 3 and Figs 2–4 and S2–S9). Semi-quantitative assessment of the immunostaining results by H-scores revealed that MUC1 was the most consistently and abundantly expressed mucin across the examined PDAC cell lines.

**Table 3:** Ratios of mucin expression [(3D + 1)/(2D + 1)] based on H-scores obtained by immunocytochemical analysis in PDAC cell lines. Expression ratios were calculated from H-scores using the formula (3D + 1)/(2D + 1). Red and blue shading indicate upregulation and downregulation in 3D culture, respectively. Asterisks denote statistical significance: *P < 0.05, **P < 0.01, and ***P < 0.001.

		Ratio (3D + 1/2D + 1)															
		MUC1		MUC2		MUC4		MUC5AC		MUC5B		MUC13		MUC19		MUC20	
**Epithelial**	**PK-8**	22.13	***	4.45	**	1.14		43.36	***	1.02		1.01		1.03		1.07	
	**PK-45P**	1.90	**	0.97		27.57	***	26.64	***	12.03	**	2.10		0.67	**	1.33	
	**PK-59**	0.82	*	4.35	**	1.33	**	41.23	***	12.51	**	10.36	***	1.35	*	1.28	
	**PK-1**	5.24	***	0.99		0.68	*	5.22	***	0.97		0.98		1.11		1.59	
	**T3M-4**	0.87		0.64		1.63	***	12.00	***	3.64	**	1.72		5.98	**	5.27	***
**Mesenchymal**	**PANC-1**	4.78	**	1.02		1.12		1.17	*	1.01		1.01		1.20		1.62	
	**KP4**	0.40	**	1.00		1.04		0.85	*	0.99		1.09		1.12		1.56	*
	**MIA PaCa-2**	1.76	***	1.00		1.01		0.80	*	1.00		1.15	*	1.04		1.88	***

**Fig 2:**
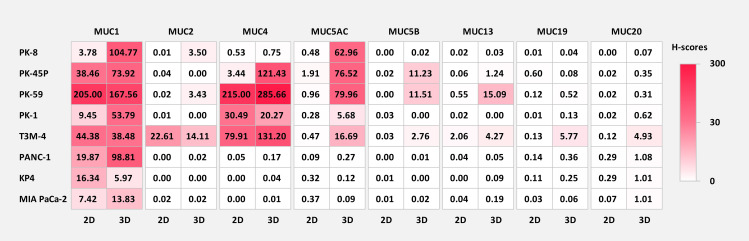
Heatmap of mucin expression in PDAC cell lines cultured under 2D and 3D conditions. This heatmap displays the H-scores of mucin expression in eight PDAC cell lines evaluated by immunocytochemical staining under 2D and 3D culture conditions. H-scores range from 0 to 300 and are visualized using a color gradient from white (low expression) to red (high expression). Each value represents the mean H-score obtained from triplicate samples in two independent experiments.

**Fig 3:**
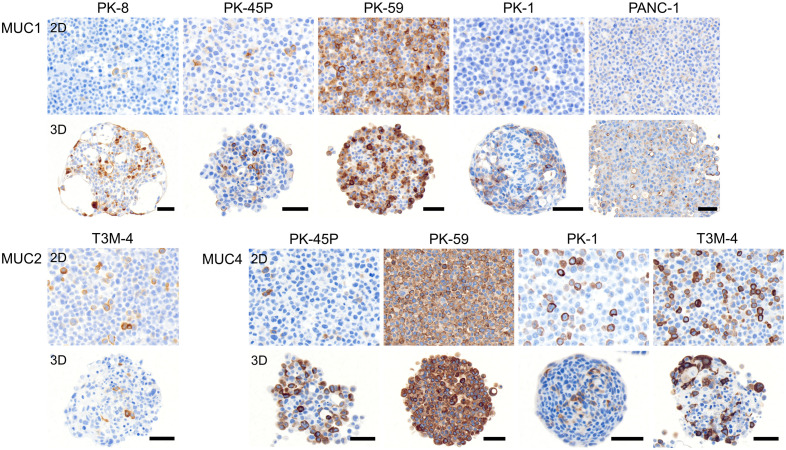
Immunocytochemical staining of MUC1, MUC2, and MUC4 in eight PDAC cell lines cultured under 2D and 3D conditions. In 2D culture, strong expression of MUC1 is evident only in PK-59 cells, whereas in 3D culture, PK-8, PK-45P, PK-59, PK-1, and PANC-1 cells show marked expression. MUC2 is detected only at low levels in a small subset of T3M-4 cells under both culture conditions. MUC4 exhibits strong staining in PK-59, PK-1, and T3M-4 cells in 2D culture, with additional high expression observed in PK-45P cells under 3D culture. Scale bars: 50 μm.

**Fig 4:**
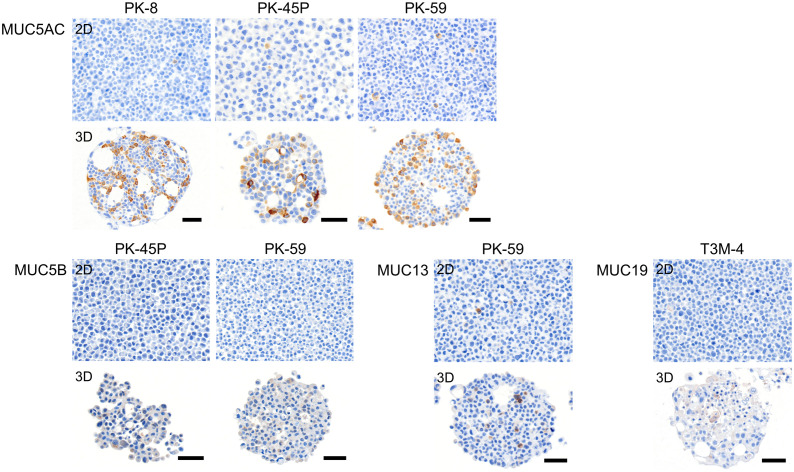
Immunocytochemical staining of MUC5AC, MUC5B, MUC13, and MUC19 in PDAC cell lines cultured under 2D and 3D conditions. MUC5AC is not detected in any pancreatic ductal adenocarcinoma (PDAC) cell lines under 2D culture but shows strong expression in PK-8, PK-45P, and PK-59 cells in 3D culture. MUC5B, MUC13, and MUC19 are absent in 2D culture. In 3D culture, scattered positive cells are observed for MUC5B in PK-45P and PK-59 cells, for MUC13 in PK-59 cells, for MUC19 in T3M-4 cells. Scale bars: 50 μm.

Under 2D culture conditions, MUC1 was positive in seven out of eight cell lines (H-score > 5, Fig 2), with PK-59 showing the highest expression level (H-score 205), while PK-8 remained negative (H-score 3.78). In 3D culture, MUC1 expression was detected in all cell lines. Strong expression (H-score ≥ 50) was observed in four epithelial lines (PK-8, PK-45P, PK-59, PK-1) and one mesenchymal line (PANC-1). MUC1 localized to both the plasma membrane and cytoplasm in epithelial PDAC cell lines, whereas it was confined to the plasma membrane in mesenchymal PDAC cell lines (Fig 3, upper panels; S2A and S2B Fig). Statistical analysis demonstrated significant upregulation of MUC1 in five cell lines under 3D culture (Table 3 and S2C Fig), although expression decreased in PK-59, which showed the highest basal level, and in KP4.

MUC2 expression was restricted to a subset of T3M-4 cells under both 2D and 3D conditions (H-score > 5, Fig 2), displaying granular cytoplasmic staining (Fig 3, lower left panels; S3A and S3B Fig). Slight but statistically significant increases were observed in PK-8 and PK-59 under 3D culture (Table 3 and S3C Fig).

MUC4 was detected in PK-59, PK-1, and T3M-4 under 2D conditions, and additionally in PK-45P under 3D conditions (H-score > 5, Fig 2). The protein localized to both the cytoplasm and membrane, with PK-59 showing strong positivity in nearly all cells under both conditions (Fig 3, lower right panels; S4A and S4B Fig). Significant upregulation was observed in PK-45P, PK-59, and T3M-4, whereas PK-1 exhibited decreased expression under 3D culture (Table 3 and S4C Fig).

MUC5AC was undetectable in all cell lines under 2D culture but was markedly induced in five epithelial PDAC cell lines under 3D conditions (H-score > 5, Fig 2). Among these, PK-8, PK-45P, and PK-59 displayed strong cytoplasmic expression (H-score ≥ 50), particularly in E-cadherin–high lines (Fig 4, upper panels; S5A and S5B Fig). Statistical analysis confirmed significant upregulation in all epithelial PDAC cell lines (Table 3 and S5C Fig). Although mesenchymal PDAC cell lines also showed statistically significant changes, all values of H-score remained within the negative range (Table 3 and Figs 2 and S5C).

MUC5B was absent in 2D culture but weakly positive (H-score 5–50, Fig 2) in PK-45P and PK-59 under 3D culture, exhibiting granular cytoplasmic staining (Fig 4, lower left panels; S6A and S6B Fig). Statistical analysis revealed increased expression in PK-45P, PK-59, and T3M-4 (Table 3 and S6C Fig).

MUC13 was negative under 2D culture but showed cytoplasmic and membranous positivity in a subset of PK-59 cells under 3D culture (Figs 2 and 4, lower middle panels; S7A and S7B Figs), with an H-score indicating weak positivity (H-score 5–50). Statistical analysis demonstrated a significant increase in MUC13 expression in PK-59 cells under 3D conditions (Table 3 and S7C Fig). In addition, MIA PaCa-2 cells also showed a statistically significant increase in expression under 3D culture; however, the H-score remained within the negative range (Table 3 and S7C Fig).

MUC19 was negative in most cells under both 2D and 3D culture conditions (Fig 2). Only T3M-4 cells under 3D culture exhibited weak positivity (H-score 5–50), showing granular staining in the cytoplasm of some cells (Fig 4, lower right panels; S8A and S8B Fig). A significant increase in MUC19 expression was observed in T3M-4 cells (Table 3 and S8C Fig). Statistically significant changes were also detected in PK-45P and PK-59 cells; however, their H-scores remained within the negative range (Table 3 and S8C Fig).

MUC20, although identified by proteomics, remained below the positivity threshold (H-score < 5) under both 2D and 3D conditions (Figs 2, S9A and S9B). Nonetheless, statistically significant upregulation was noted in T3M-4, KP4, and MIA PaCa-2, again within the negative range (Table 3 and S9C Fig).

Collectively, these findings indicate that all PDAC cell lines expressed MUC1 in 3D culture, whereas epithelial-type lines exhibited 3D-induced expression of MUC5AC, MUC5B, MUC13, and MUC19 (Table 3 and Fig 2). In contrast, mesenchymal-type PDAC cell lines expressed only MUC1, highlighting the distinct mucin expression profiles between epithelial and mesenchymal phenotypes.

## Discussion

In this study, eight mucins (MUC1, MUC2, MUC4, MUC5AC, MUC5B, MUC13, MUC19, and MUC20) identified by proteomic analysis were examined in eight PDAC cell lines by immunocytochemical analysis, and their expression was semi-quantitatively assessed using the H-score system. Epithelial PDAC cell lines exhibited extensive reorganization of mucin expression under 3D culture, with particularly pronounced changes in MUC1, MUC4, and MUC5AC. In contrast, mesenchymal lines displayed alterations mainly in MUC1, while changes in other mucins were limited. To our knowledge, this represents the first systematic comparison of mucin expression across epithelial and mesenchymal PDAC cell lines under 2D and 3D culture conditions, highlighting the plasticity of mucin expression in response to microenvironmental cues.

Proteomic analysis comprehensively characterized mucin expression in all PDAC cell lines. Comparison of protein sequence coverage, total peptide counts, and unique peptide counts between 2D and 3D conditions revealed that all mucins showed similar or increased detection in 3D culture. Notably, MUC5AC was undetectable under 2D conditions but was identified by multiple peptides under 3D culture. Although H-scores and peptide detection numbers generally correlated, MUC2 and MUC19 showed low unique peptide counts (0 and 1, respectively), suggesting limited reliability relative to other mucins. The overall coverage of most mucins, except MUC20, was below 10%, consistent with the technical challenges of analyzing highly glycosylated, high-molecular-weight proteins. These discrepancies likely reflect the structural complexity of mucins, which restrict digestion efficiency, peptide recovery, and ionization sensitivity [27]. Methodological differences between membrane-associated mucins (MUC1, MUC4, MUC13, MUC20) and secretory mucins (MUC2, MUC5AC, MUC5B, MUC19) may also contribute. Because 3D culture can modify polarity, secretory pathways, and glycosylation patterns, accurate mucin profiling may require complementary approaches such as membrane–secretory fractionation, deglycosylation pretreatment, antibody clone validation, and standardized image analysis.

Immunocytochemistry confirmed MUC1 expression in all epithelial and mesenchymal PDAC cell lines under 3D culture. MUC1 is associated with invasive growth and poor prognosis and to promote a proangiogenic microenvironment via the neuropilin-1 and its ligand, vascular endothelial growth factor [28]. Its cytoplasmic tail interacts with multiple signaling pathways, including Ras, β-catenin, p53, and estrogen receptor α [29]. Genetic or pharmacological inhibition of MUC1 suppresses proliferation, migration, and invasion of PDAC cells, enhances apoptosis, and increases sensitivity to gemcitabine and 5-fluorouracil [30]. Clinical trials of MUC1-targeted vaccines and monoclonal antibodies for pancreatic cancer are ongoing [31–35]. In our study, MUC1 expression was enhanced under 3D culture in PK-8, PK-45P, PK-1, PANC-1, and MIA PaCa-2 cells, whereas PK-59 and KP4 showed decreased levels. However, MUC1 remained strongly positive in PK-59 and weakly positive in KP4 under 3D conditions, indicating that these decreases reflect quantitative variation rather than loss of expression. The reduced H-score in PK-59 may be attributable to 3D culture-associated changes in cellular organization, microenvironmental gradients, glycosylation, or epitope accessibility that influence immunocytochemical detection [19,20,36]. Therefore, MUC1 should be regarded as a stable but quantitatively variable marker across PDAC phenotypes and culture conditions.

MUC5AC, a gel-forming mucin that contributes to mucus viscoelasticity, also plays an important role in PDAC biology. Previous knockdown studies have demonstrated that suppression of MUC5AC reduces tumorigenicity and in vivo growth and modulates TRAIL-induced apoptosis [37,38]. In this study, MUC5AC expression was consistently upregulated under 3D culture in all epithelial-type cell lines, suggesting that its induction reflects alterations in the secretory microenvironment. By contrast, mesenchymal-type lines exhibited only modest changes, implying constraints associated with their differentiation status and morphology. The observed >5-fold increase in H-scores across epithelial lines highlights the potential of MUC5AC as a microenvironment-sensitive biomarker.

MUC4 has been reported to promote proliferation, invasion, and survival of PDAC cells through inhibition of apoptotic signaling and induction of epithelial–mesenchymal transition [39–41]. It also contributes to gemcitabine resistance, and MUC4 suppression enhances chemosensitivity [42,43]. In our study, MUC4 expression increased under 3D culture in PK-45P, PK-59, and T3M-4 cells, with PK-59 spheres showing uniformly strong staining across the entire cell population. These results further emphasize that MUC4 expression is highly microenvironment-dependent and may directly influence therapeutic responsiveness.

Conventional 2D culture fails to recapitulate the morphological heterogeneity and substrate-dependent signaling of tumor cells [44]. By contrast, 3D culture reconstructs structural architecture and microenvironmental gradients, thereby amplifying functional heterogeneity. Consistent with this, mucins such as MUC2, MUC5AC, MUC5B, MUC13, and MUC19, which were minimally expressed under 2D conditions, were markedly upregulated under 3D conditions, particularly in epithelial-type cell lines. These changes are likely driven by extracellular matrix signaling, hypoxia, TGF-β pathway activation, and epigenetic regulation, including DNA methylation and histone modification [45].

Clinically, mucin expression profiles are used to distinguish PDAC from IPMN-derived carcinomas—for example, gastric type (MUC5AC-dominant), intestinal type (MUC2 + /MUC5AC+), and pancreatobiliary type (MUC1 + /MUC5AC+ with MUC2−) [46–48]. Although this study was limited to PDAC cell lines, 3D culture induced MUC5AC and occasionally MUC2 expression, whereas MUC1 remained widely expressed across conditions. These findings suggest that reliance on single mucin markers may lead to diagnostic misinterpretation. Based on the present H-score data, a panel combining MUC1, MUC5AC, and MUC4 may be particularly useful for differentiating PDAC phenotypes under 3D conditions. MUC1 was widely expressed across PDAC cell lines, whereas MUC5AC and MUC4 were preferentially induced or maintained in epithelial-type cell lines. In contrast, mesenchymal-type cell lines retained MUC1 expression but showed limited expression of MUC5AC and MUC4. Thus, MUC1/MUC5AC/MUC4 may represent a candidate mucin panel for future validation in patient-derived organoids and tissue-based studies. Since mucin expression is both plastic and microenvironment-dependent, pathological interpretation should consider epithelial–mesenchymal phenotype, subcellular localization, microenvironmental context, and temporal variation, employing a panel-based diagnostic approach.

This study has some limitations. First, our analyses were based on a limited panel of eight established PDAC cell lines. Although this panel included both epithelial-classical and mesenchymal-quasi-mesenchymal phenotypes and provided internally consistent evidence of 3D culture-associated remodeling of mucin expression, it does not fully represent the molecular and phenotypic diversity of PDAC [10,11,13]. Therefore, validation using larger cell-line panels, patient-derived organoids, and clinical tissue specimens are required.

Second, 3D culture conditions may influence mucin expression. In the present study, all cell lines were cultured under the same spheroid-forming protocol; however, differences in matrix composition, oxygen gradients, culture duration, medium exchange, and spheroid compactness may alter the magnitude of mucin reorganization [20,49]. Future studies should standardize these parameters and evaluate their effects systematically.

Third, both proteomic and immunocytochemical analyses have inherent technical constraints. Proteomic detection of mucins may be affected by low sequence coverage, peptide sharing among mucin family members, and limited detection sensitivity for heavily glycosylated proteins. Immunocytochemical evaluation may also be influenced by antibody specificity, glycosylation-dependent epitope masking, and semi-quantitative scoring.

Future research should integrate optimized 3D culture systems with co-culture models incorporating stromal and immune components and validate findings using patient-derived tissues and organoids. In addition, experimental interventions targeting signaling pathways and epigenetic regulators will be essential to elucidate the molecular mechanisms governing mucin plasticity. Ultimately, these approaches will provide a foundation for translational applications, including biomarker panel development and mucin-targeted therapeutic strategies for PDAC.

## Conclusions

In conclusion, this study demonstrates that 3D culture profoundly reorganizes mucin expression programs in PDAC cell lines, notably enhancing MUC1, MUC5AC, and MUC4 expression in epithelial PDAC cell lines while maintaining restricted profiles in mesenchymal lines. These results highlight the dynamic and microenvironment-dependent nature of mucin regulation, highlighting the potential limitations of single-marker–based diagnosis and emphasizing the value of comprehensive, panel-based approaches. Although this study was conducted using established cell lines, our findings provide key insights into the diagnostic, subclassifying, and therapeutic implications of mucins in PDAC. Future investigations integrating optimized 3D culture systems with patient-derived materials will be essential to elucidate the clinical significance of mucin plasticity and to promote its translational application in precision oncology.

## Supporting information

S1 Fig H-score measurement method. (DOCX)

S2 Fig Localization, expression level, and statistical comparison of MUC1 in 2D- and 3D-cultured PDAC cell lines. (DOCX)

S3 Fig Localization, expression level, and statistical comparison of MUC2 in 2D- and 3D-cultured PDAC cell lines. (DOCX)

S4 Fig Localization, expression level, and statistical comparison of MUC4 in 2D- and 3D-cultured PDAC cell lines. (DOCX)

S5 Fig Localization, expression level, and statistical comparison of MUC5AC in 2D- and 3D-cultured PDAC cell lines. (DOCX)

S6 Fig Localization, expression level, and statistical comparison of MUC5B in 2D- and 3D-cultured PDAC cell lines. (DOCX)

S7 Fig Localization, expression level, and statistical comparison of MUC13 in 2D- and 3D-cultured PDAC cell lines. (DOCX)

S8 Fig Localization, expression level, and statistical comparison of MUC19 in 2D- and 3D-cultured PDAC cell lines. (DOCX)

S9 Fig Localization, expression level, and statistical comparison of MUC20 in 2D- and 3D-cultured PDAC cell lines. (DOCX)

S1 Table Statistical results for comparisons of mucin H-scores between 2D- and 3D-cultured pancreatic ductal adenocarcinoma cell lines before and after Benjamini–Hochberg false discovery rate correction. (DOCX)
