# Supplementary material for: Mucin expression in pancreatic ductal adenocarcinoma cell lines in 2D and 3D cultures: A proteomic and immunocytochemical analysis
Source: PLoS One. 2026 Jul 16;21(7):e0353991. doi: 10.1371/journal.pone.0353991 (PMC13374910; doi:10.1371/journal.pone.0353991)
Supplement: S1 Fig — (DOCX) [file pone.0353991.s001.docx]

**
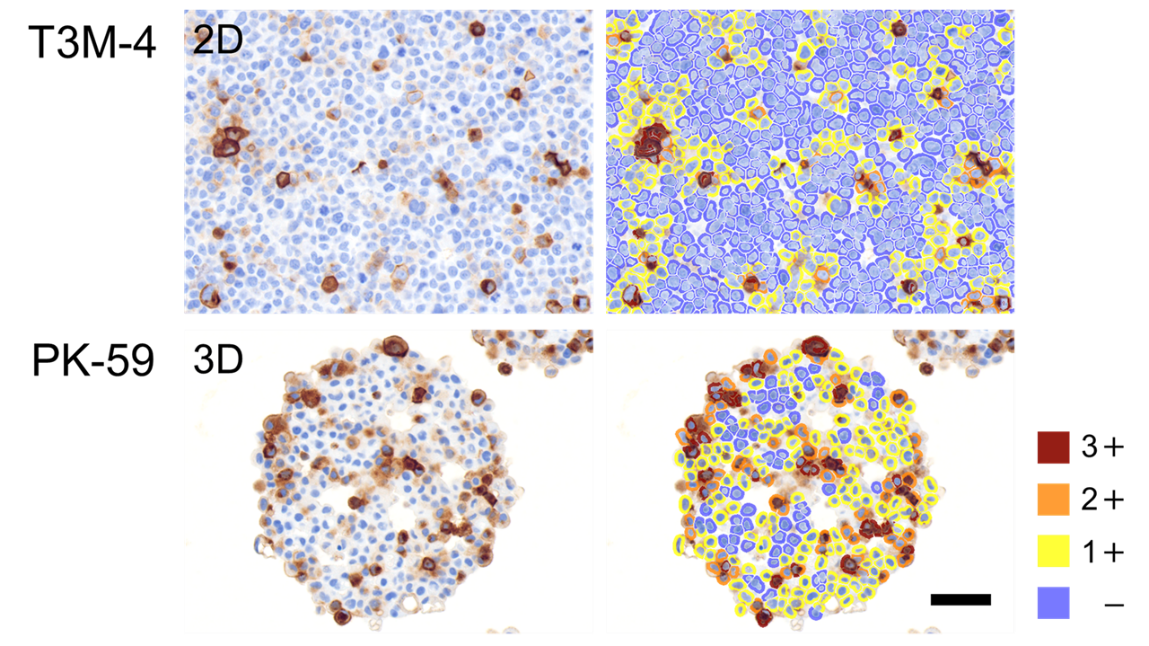
**

**S1 Fig. H-score measurement method**

The intensity is divided into four categories: negative, weakly positive, positive, and strongly positive, with scores of 0, 1, 2, and 3, respectively. The H-score is calculated by multiplying the percentage of cells at each intensity. Scale bar, 50 µm.

H-score (2D): 0 × 68.58 % + 1× 23.97 % + 2 × 3.65 % + 3×3.81 % = 42.70

H-score (3D): 0 × 26.87 % + 1 × 40.48 % + 2 × 18.37 % + 3 × 14.29 % = 120.09
