# Supplementary material for: Mucin expression in pancreatic ductal adenocarcinoma cell lines in 2D and 3D cultures: A proteomic and immunocytochemical analysis
Source: PLoS One. 2026 Jul 16;21(7):e0353991. doi: 10.1371/journal.pone.0353991 (PMC13374910; doi:10.1371/journal.pone.0353991)
Supplement: S5 Fig — (DOCX) [file pone.0353991.s005.docx]

**
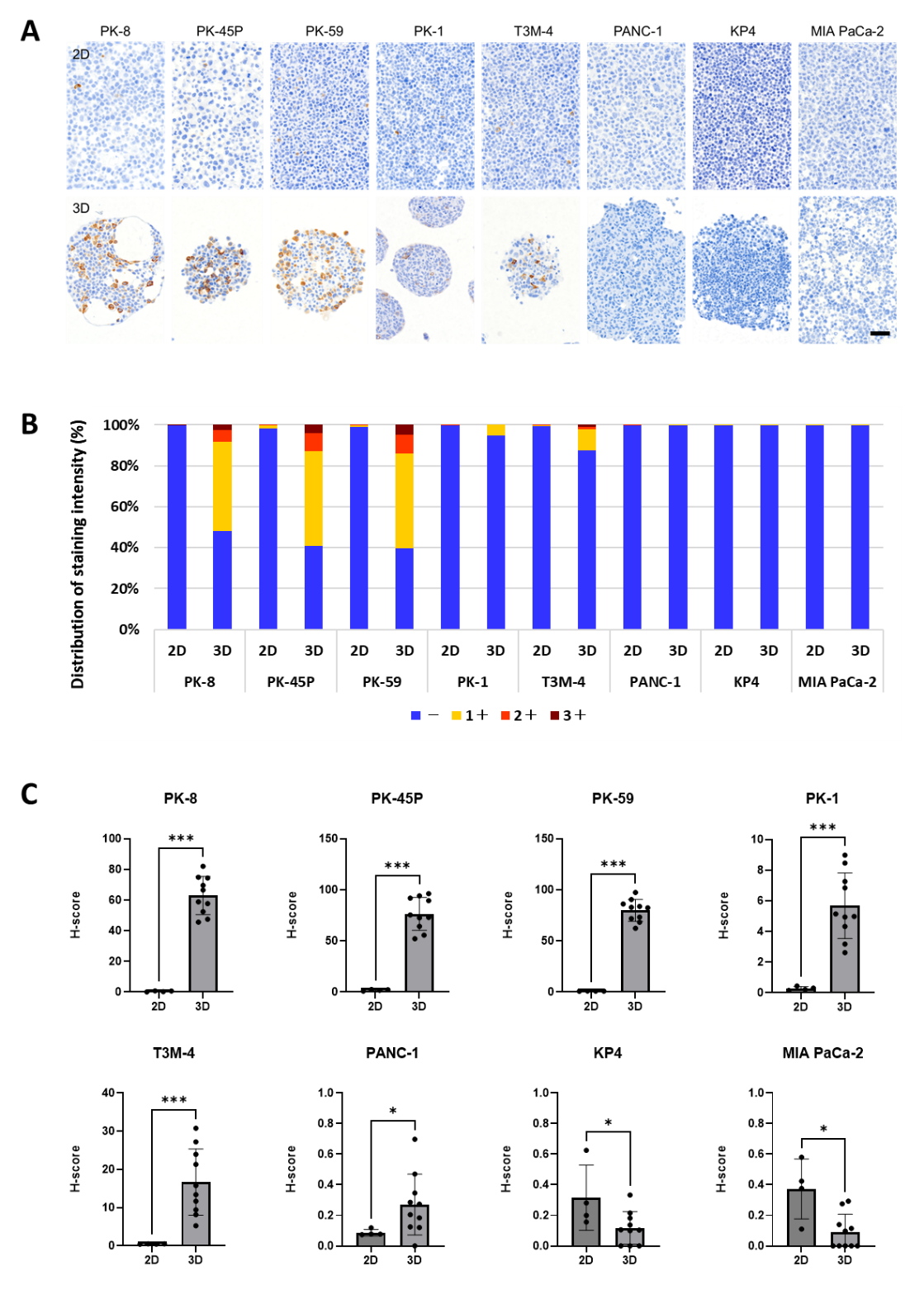
**

**S5 Fig. Localization, expression level, and statistical comparison of MUC5AC in 2D- and 3D-cultured PDAC cell lines**

(A) Representative immunocytochemical images show the localization of MUC5AC in PDAC cell lines cultured under 2D and 3D conditions. All PDAC cell lines are negative under 2D conditions, whereas all epithelial PDAC cell lines become positive under 3D culture (H-score > 5). (B) Bar graph illustrates the percentage of MUC5AC-positive cells classified by staining intensity (−, 1+, 2+, 3+). (C) Comparison of H-scores between 2D and 3D cultures in eight PDAC cell lines. Data are presented as mean ± SD; **P* < 0.05, ****P* < 0.001. Scale bar, 50 µm.
