# Supplementary material for: Mucin expression in pancreatic ductal adenocarcinoma cell lines in 2D and 3D cultures: A proteomic and immunocytochemical analysis
Source: PLoS One. 2026 Jul 16;21(7):e0353991. doi: 10.1371/journal.pone.0353991 (PMC13374910; doi:10.1371/journal.pone.0353991)
Supplement: S6 Fig — (DOCX) [file pone.0353991.s006.docx]

**
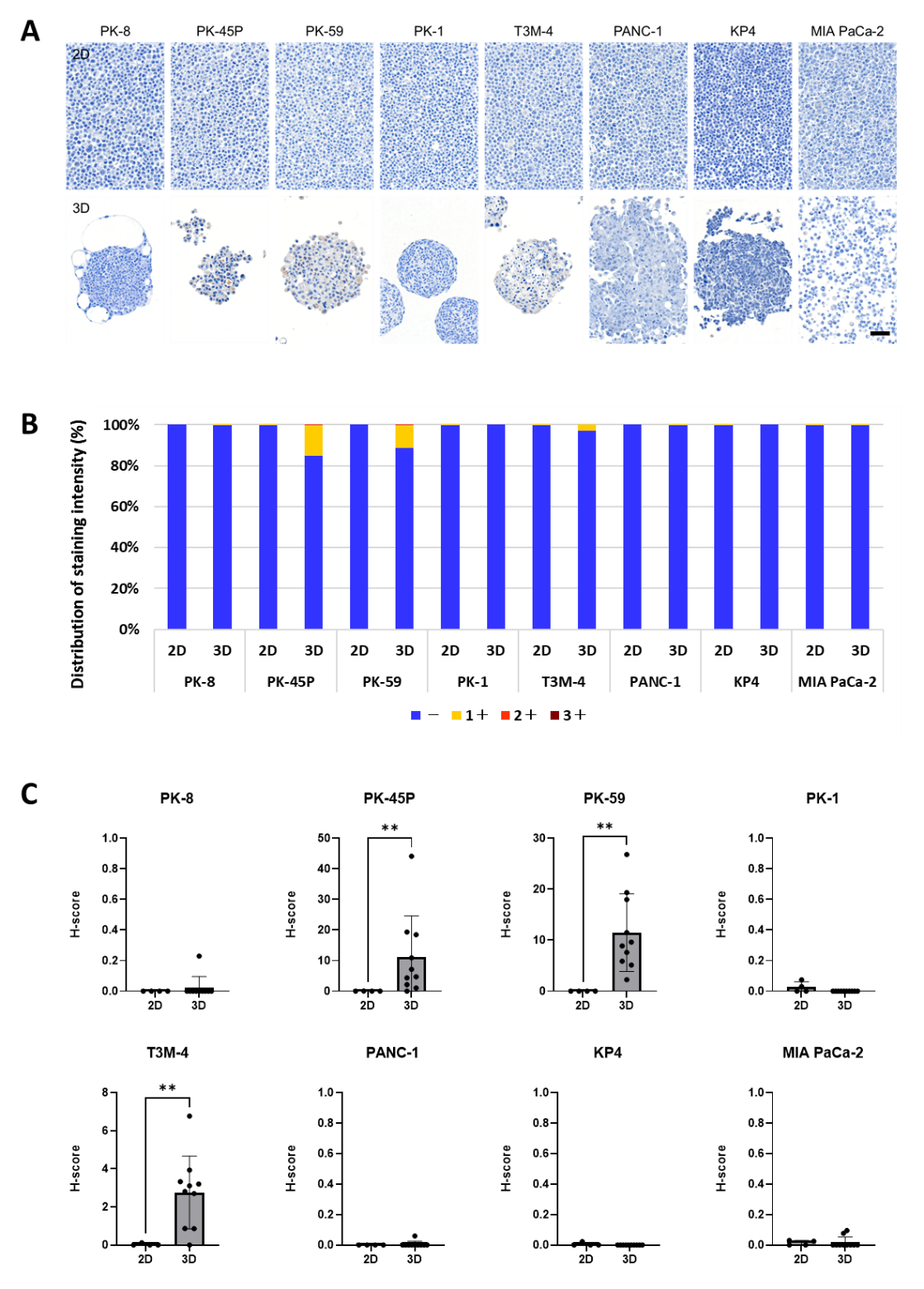
**

**S6 Fig. Localization, expression level, and statistical comparison of MUC5B in 2D- and 3D-cultured PDAC cell lines**

(A) Representative immunocytochemical images show the localization of MUC5B in PDAC cell lines cultured under 2D and 3D conditions. PK-45P and PK-59 cells become positive under 3D culture (H-score > 5). (B) Bar graph illustrates the percentage of MUC5B-positive cells classified by staining intensity (−, 1+, 2+, 3+). (C) Comparison of H-scores between 2D and 3D cultures in eight PDAC cell lines. Data are presented as mean ± SD; ***P* < 0.01. Scale bar, 50 µm.
