# Supplementary material for: Mucin expression in pancreatic ductal adenocarcinoma cell lines in 2D and 3D cultures: A proteomic and immunocytochemical analysis
Source: PLoS One. 2026 Jul 16;21(7):e0353991. doi: 10.1371/journal.pone.0353991 (PMC13374910; doi:10.1371/journal.pone.0353991)
Supplement: S9 Fig — (DOCX) [file pone.0353991.s009.docx]

**
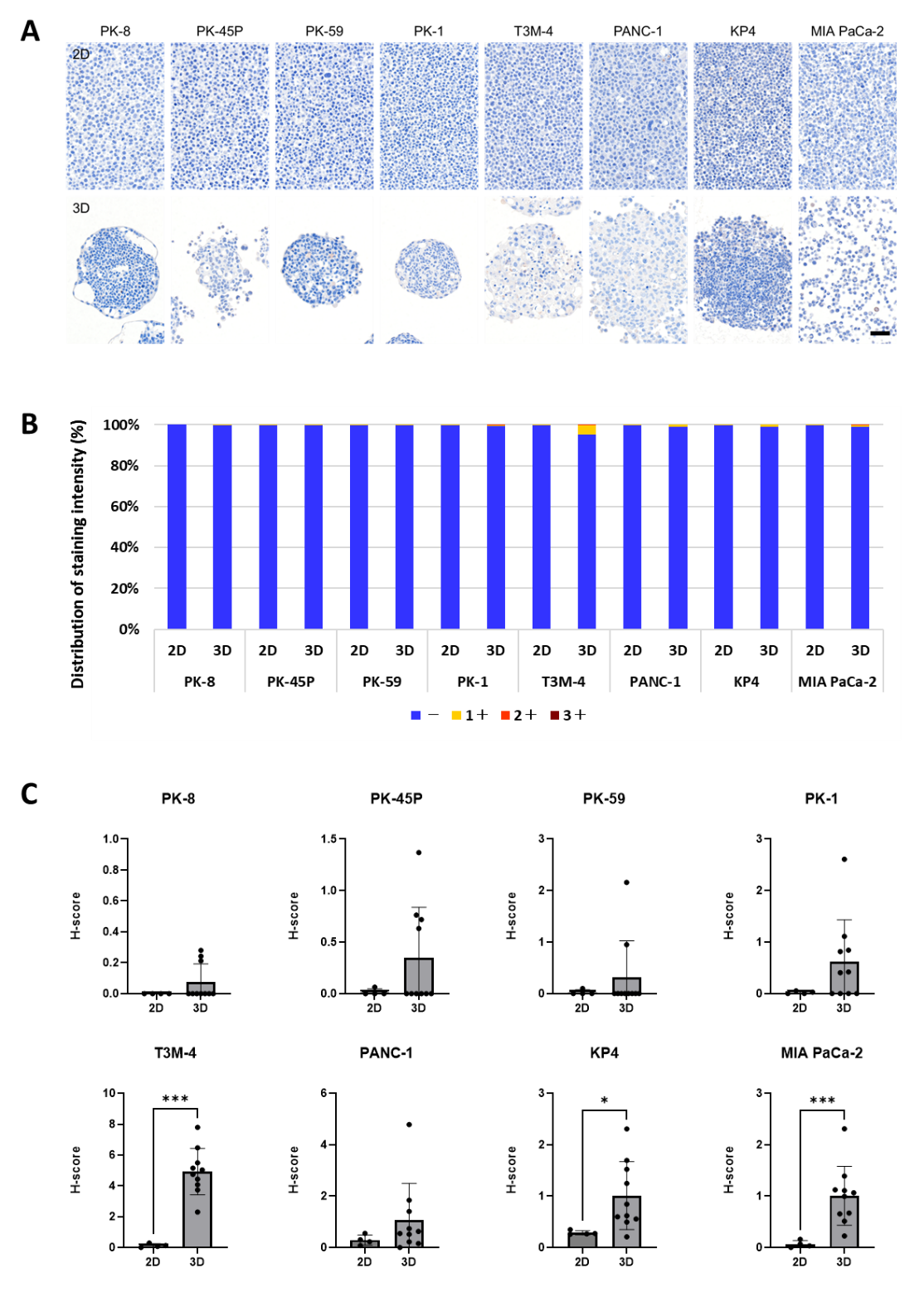
**

**S9 Fig. Localization, expression level, and statistical comparison of MUC20 in 2D- and 3D-cultured PDAC cell lines**

(A) Representative immunocytochemical images show the localization of MUC20 in PDAC cell lines cultured under 2D and 3D conditions. MUC20 is negative under both conditions (H-score < 5). (B) Bar graph illustrates the percentage of MUC20-positive cells classified by staining intensity (−, 1+, 2+, 3+). (C) Comparison of H-scores between 2D and 3D cultures in eight PDAC cell lines. Data are presented as mean ± SD; **P* < 0.05, ****P* < 0.01. Scale bar, 50 µm.
