# Supplementary material for: Mucin expression in pancreatic ductal adenocarcinoma cell lines in 2D and 3D cultures: A proteomic and immunocytochemical analysis
Source: PLoS One. 2026 Jul 16;21(7):e0353991. doi: 10.1371/journal.pone.0353991 (PMC13374910; doi:10.1371/journal.pone.0353991)
Supplement: S1 Table — (DOCX) [file pone.0353991.s010.docx]

**S1 Table. Statistical results for comparisons of mucin H-scores between 2D- and 3D-cultured pancreatic ductal adenocarcinoma cell lines before and after Benjamini–Hochberg false discovery rate (FDR) correction**

Raw *P* values were obtained using the statistical test indicated in the “Test used” column (unpaired t-test, Welch’s t-test, or Mann–Whitney U test). Benjamini–Hochberg FDR correction was subsequently applied across all mucin–cell line comparisons. Adjusted q values < 0.05 were considered statistically significant after FDR correction. Difference was calculated as mean H-score in 2D culture minus mean H-score in 3D culture. The symbol † indicates comparisons that were statistically significant in the primary analysis based on the raw *P* value (*P* < 0.05) but did not remain significant after Benjamini–Hochberg correction (adjusted q value ≥ 0.05).

| **Mucin** | **Cell line** | **Test used** | **2D H-score (mean ± SD)** | **3D H-score (mean ± SD)** | **Difference  (2D - 3D)** | **Raw  P value** | **Adjusted  q value** | **Significant after FDR** |
| --- | --- | --- | --- | --- | --- | --- | --- | --- |
| MUC1 | PK-8 | Welch's t-test | 3.78 ± 0.77 | 104.77 ± 14.45 | -100.99 | <0.000001 | <0.000011 | Yes |
| MUC1 | PK-45P | Mann-Whitney U test | 38.46 ± 7.34 | 73.92 ± 10.60 | -35.46 | 0.001998 | 0.007104 | Yes |
| MUC1 | PK-59 | Unpaired t-test | 205.00 ± 19.06 | 167.56 ± 24.35 | 37.44 | 0.018085 | 0.044517 | Yes |
| MUC1 | PK-1 | Unpaired t-test | 9.45 ± 3.28 | 53.79 ± 11.79 | -44.34 | 0.000010 | 0.000071 | Yes |
| MUC1 | T3M-4 | Mann-Whitney U test | 44.38 ± 11.15 | 38.48 ± 4.97 | 5.90 | 0.453546 | 0.617595 | No |
| MUC1 | PANC-1 | Mann-Whitney U test | 19.87 ± 4.49 | 98.81 ± 1.34 | -78.95 | 0.001998 | 0.007104 | Yes |
| MUC1 | KP4 | Mann-Whitney U test | 16.34 ± 5.37 | 5.97 ± 3.16 | 10.37 | 0.003996 | 0.012787 | Yes |
| MUC1 | MIA PaCa-2 | Unpaired t-test | 7.42 ± 1.21 | 13.83 ± 2.24 | -6.41 | 0.000180 | 0.001047 | Yes |
| MUC2 | PK-8 | Mann-Whitney U test | 0.01 ± 0.02 | 3.50 ± 1.93 | -3.49 | 0.001998 | 0.007104 | Yes |
| MUC2 | PK-45P | Mann-Whitney U test | 0.04 ± 0.07 | 0.00 ± 0.00 | 0.04 | 0.285714 | 0.425249 | No |
| MUC2 | PK-59 | Mann-Whitney U test | 0.02 ± 0.04 | 3.43 ± 1.30 | -3.41 | 0.001998 | 0.007104 | Yes |
| MUC2 | PK-1 | Mann-Whitney U test | 0.01 ± 0.02 | 0.00 ± 0.00 | 0.01 | 0.285714 | 0.425249 | No |
| MUC2 | T3M-4 | Mann-Whitney U test | 22.61 ± 4.13 | 14.11 ± 8.99 | 8.50 | 0.075924 | 0.142916 | No |
| MUC2 | PANC-1 | Mann-Whitney U test | 0.00 ± 0.00 | 0.02 ± 0.06 | -0.02 | >0.999999 | >0.999999 | No |
| MUC2 | KP4 | Mann-Whitney U test | 0.00 ± 0.00 | 0.00 ± 0.00 | 0.00 | >0.999999 | >0.999999 | No |
| MUC2 | MIA PaCa-2 | Mann-Whitney U test | 0.02 ± 0.02 | 0.02 ± 0.03 | 0.00 | >0.999999 | >0.999999 | No |
| MUC4 | PK-8 | Unpaired t-test | 0.53 ± 0.31 | 0.75 ± 0.45 | -0.22 | 0.400600 | 0.569742 | No |
| MUC4 | PK-45P | Welch's t-test | 3.44 ± 0.59 | 121.43 ± 18.09 | -117.99 | <0.000001 | <0.000011 | Yes |
| MUC4 | PK-59 | Welch's t-test | 215.00 ± 21.48 | 285.66 ± 4.41 | -70.66 | 0.006593 | 0.019180 | Yes |
| MUC4 | PK-1 | Unpaired t-test | 30.49 ± 3.25 | 20.27 ± 7.73 | 10.23 | 0.027463 | 0.058588 | No^†^ |
| MUC4 | T3M-4 | Welch's t-test | 79.91 ± 4.96 | 131.20 ± 19.35 | -51.30 | 0.000007 | 0.000056 | Yes |
| MUC4 | PANC-1 | Mann-Whitney U test | 0.05 ± 0.04 | 0.17 ± 0.23 | -0.12 | 0.730270 | 0.865505 | No |
| MUC4 | KP4 | Mann-Whitney U test | 0.00 ± 0.00 | 0.04 ± 0.10 | -0.04 | 0.560440 | 0.689772 | No |
| MUC4 | MIA PaCa-2 | Mann-Whitney U test | 0.00 ± 0.00 | 0.01 ± 0.03 | -0.01 | >0.999999 | >0.999999 | No |
| MUC5AC | PK-8 | Welch's t-test | 0.48 ± 0.33 | 62.96 ± 12.43 | -62.49 | <0.000001 | <0.000011 | Yes |
| MUC5AC | PK-45P | Welch's t-test | 1.91 ± 0.70 | 76.52 ± 16.03 | -74.61 | <0.000001 | <0.000011 | Yes |
| MUC5AC | PK-59 | Welch's t-test | 0.96 ± 0.08 | 79.96 ± 10.75 | -79.00 | <0.000001 | <0.000011 | Yes |
| MUC5AC | PK-1 | Welch's t-test | 0.28 ± 0.13 | 5.68 ± 2.14 | -5.40 | 0.000021 | 0.000134 | Yes |
| MUC5AC | T3M-4 | Welch's t-test | 0.47 ± 0.05 | 16.69 ± 8.67 | -16.22 | 0.000225 | 0.001200 | Yes |
| MUC5AC | PANC-1 | Mann-Whitney U test | 0.09 ± 0.02 | 0.27 ± 0.20 | -0.18 | 0.023976 | 0.052913 | No^†^ |
| MUC5AC | KP4 | Unpaired t-test | 0.32 ± 0.21 | 0.12 ± 0.11 | 0.20 | 0.035234 | 0.072741 | No^†^ |
| MUC5AC | MIA PaCa-2 | Mann-Whitney U test | 0.37 ± 0.20 | 0.09 ± 0.12 | 0.28 | 0.016983 | 0.043476 | Yes |
| MUC5B | PK-8 | Mann-Whitney U test | 0.00 ± 0.00 | 0.02 ± 0.07 | -0.02 | >0.999999 | >0.999999 | No |
| MUC5B | PK-45P | Mann-Whitney U test | 0.02 ± 0.03 | 11.23 ± 13.42 | -11.21 | 0.008991 | 0.023976 | Yes |
| MUC5B | PK-59 | Mann-Whitney U test | 0.00 ± 0.00 | 11.51 ± 7.61 | -11.51 | 0.001998 | 0.007104 | Yes |
| MUC5B | PK-1 | Mann-Whitney U test | 0.03 ± 0.04 | 0.00 ± 0.00 | 0.03 | 0.065934 | 0.127872 | No |
| MUC5B | T3M-4 | Mann-Whitney U test | 0.03 ± 0.06 | 2.76 ± 1.91 | -2.73 | 0.008991 | 0.023976 | Yes |
| MUC5B | PANC-1 | Mann-Whitney U test | 0.00 ± 0.00 | 0.01 ± 0.02 | -0.01 | >0.999999 | >0.999999 | No |
| MUC5B | KP4 | Mann-Whitney U test | 0.01 ± 0.01 | 0.00 ± 0.00 | 0.01 | 0.285714 | 0.425249 | No |
| MUC5B | MIA PaCa-2 | Mann-Whitney U test | 0.01 ± 0.02 | 0.02 ± 0.04 | 0.00 | 0.520480 | 0.666214 | No |
| MUC13 | PK-8 | Mann-Whitney U test | 0.02 ± 0.03 | 0.03 ± 0.10 | -0.02 | >0.999999 | >0.999999 | No |
| MUC13 | PK-45P | Mann-Whitney U test | 0.06 ± 0.06 | 1.24 ± 1.78 | -1.17 | 0.190809 | 0.321363 | No |
| MUC13 | PK-59 | Welch's t-test | 0.55 ± 0.31 | 15.09 ± 4.20 | -14.54 | <0.000001 | <0.000011 | Yes |
| MUC13 | PK-1 | Mann-Whitney U test | 0.02 ± 0.04 | 0.00 ± 0.00 | 0.02 | 0.285714 | 0.425249 | No |
| MUC13 | T3M-4 | Unpaired t-test | 2.06 ± 1.14 | 4.27 ± 2.83 | -2.21 | 0.164108 | 0.291748 | No |
| MUC13 | PANC-1 | Mann-Whitney U test | 0.04 ± 0.05 | 0.05 ± 0.09 | -0.01 | >0.999999 | >0.999999 | No |
| MUC13 | KP4 | Mann-Whitney U test | 0.00 ± 0.00 | 0.09 ± 0.23 | -0.09 | 0.560440 | 0.689772 | No |
| MUC13 | MIA PaCa-2 | Welch's t-test | 0.04 ± 0.04 | 0.19 ± 0.17 | -0.15 | 0.040120 | 0.080240 | No^†^ |
| MUC19 | PK-8 | Mann-Whitney U test | 0.01 ± 0.02 | 0.04 ± 0.12 | -0.03 | >0.999999 | >0.999999 | No |
| MUC19 | PK-45P | Mann-Whitney U test | 0.60 ± 0.11 | 0.08 ± 0.25 | 0.53 | 0.004995 | 0.015223 | Yes |
| MUC19 | PK-59 | Welch's t-test | 0.12 ± 0.08 | 0.52 ± 0.44 | -0.39 | 0.021543 | 0.051065 | No^†^ |
| MUC19 | PK-1 | Mann-Whitney U test | 0.01 ± 0.02 | 0.13 ± 0.40 | -0.11 | 0.175824 | 0.304128 | No |
| MUC19 | T3M-4 | Welch's t-test | 0.13 ± 0.12 | 5.77 ± 4.51 | -5.64 | 0.003345 | 0.011267 | Yes |
| MUC19 | PANC-1 | Mann-Whitney U test | 0.14 ± 0.08 | 0.36 ± 0.61 | -0.22 | 0.623377 | 0.752757 | No |
| MUC19 | KP4 | Unpaired t-test | 0.11 ± 0.07 | 0.25 ± 0.16 | -0.14 | 0.128901 | 0.235705 | No |
| MUC19 | MIA PaCa-2 | Mann-Whitney U test | 0.03 ± 0.03 | 0.06 ± 0.07 | -0.04 | 0.517483 | 0.666214 | No |
| MUC20 | PK-8 | Mann-Whitney U test | 0.00 ± 0.00 | 0.07 ± 0.12 | -0.07 | 0.505495 | 0.666214 | No |
| MUC20 | PK-45P | Mann-Whitney U test | 0.02 ± 0.03 | 0.35 ± 0.49 | -0.33 | 0.448551 | 0.617595 | No |
| MUC20 | PK-59 | Mann-Whitney U test | 0.02 ± 0.05 | 0.31 ± 0.71 | -0.29 | >0.999999 | >0.999999 | No |
| MUC20 | PK-1 | Mann-Whitney U test | 0.02 ± 0.03 | 0.62 ± 0.81 | -0.60 | 0.258741 | 0.424601 | No |
| MUC20 | T3M-4 | Welch's t-test | 0.12 ± 0.13 | 4.93 ± 1.50 | -4.80 | 0.000003 | 0.000027 | Yes |
| MUC20 | PANC-1 | Mann-Whitney U test | 0.29 ± 0.20 | 1.08 ± 1.42 | -0.79 | 0.303696 | 0.441740 | No |
| MUC20 | KP4 | Mann-Whitney U test | 0.29 ± 0.04 | 1.01 ± 0.66 | -0.72 | 0.023976 | 0.052913 | No^†^ |
| MUC20 | MIA PaCa-2 | Welch's t-test | 0.07 ± 0.07 | 1.01 ± 0.57 | -0.94 | 0.000528 | 0.002599 | Yes |
