# Supplementary material for: Mucin expression in pancreatic ductal adenocarcinoma cell lines in 2D and 3D cultures: A proteomic and immunocytochemical analysis
Source: PLoS One. 2026 Jul 16;21(7):e0353991. doi: 10.1371/journal.pone.0353991 (PMC13374910; doi:10.1371/journal.pone.0353991)
Supplement: Attachment — Submitted filename: 6-15-2026__Response_to_Reviewers_for submission.docx [file pone.0353991.s012.docx]

**Response to Reviewers**

Manuscript ID: PONE-D-25-58221

*Mucin expression in pancreatic ductal adenocarcinoma cell lines in 2D and 3D cultures: a proteomic and immunocytochemical analysis*

Dear Dr. Choudhery and Reviewers,

We sincerely thank the Academic Editor and the two reviewers for their thoughtful and constructive evaluation of our manuscript. We were encouraged that both reviewers judged the work to be technically sound, statistically appropriate, with fully available data, and clearly written. We are also grateful for the reviewers’ insightful comments and suggestions, which have helped us improve the clarity, methodological transparency, and interpretation of the study.

We have addressed every point below. Reviewer comments are reproduced in italics, followed by our responses. The corresponding revisions have been incorporated into the manuscript and highlighted in the tracked-changes version. Page and line numbers refer to the revised manuscript.

## Responses to Reviewer #1

We thank Reviewer #1 for their careful review of our manuscript and the seventeen specific suggestions, which have substantially strengthened the manuscript.

**Comment 1.**

*Authors state in the Introduction that 3D culture better reflects tissue architecture, but they do not explain why this would be the case for mucins. Why do Authors expect changes in mucin expression upon transition from 2D to 3D?*

**Response:**

Thank you for this important comment. We have revised the Introduction (Page 4, Lines 71–80) to clarify the biological rationale for comparing mucin expression between 2D and 3D cultures. Specifically, we now explain that 3D spheroids differ from 2D monolayers in cell–cell adhesion, epithelial polarity, cell density, and oxygen/nutrient gradients, all of which may influence mucin biosynthesis, glycosylation, trafficking, and secretion. We therefore present mucin remodeling as a hypothesis arising from architectural and microenvironmental differences between culture systems.

Page 4, Lines 71–80: “Mucins may be particularly susceptible to remodeling during the transition from 2D to 3D culture. This is because mucin biosynthesis depends on epithelial polarity, Golgi-resident mucin-type O-glycosylation, and polarized secretory trafficking [15-17]. These epithelial features are not fully recapitulated in flat monolayer cultures, whereas 3D spheres can partially restore tissue-like architecture, cell–cell interactions, and apical–basal polarity, while also generating gradients of oxygen and nutrients [18-20]. In addition, hypoxic and stress-responsive microenvironments in 3D cultures may contribute to the up-regulation of selected membrane-tethered and gel-forming mucins through HIF-1α-dependent pathways and endoplasmic reticulum stress or unfolded protein response signaling [21-24].”

**Comment 2.**

*Authors state their research hypothesis regarding proteomic analysis and ICC. Will these analyses focus on the protein core or specific glycans? This is worth clarifying in the Introduction.*

**Response:**

We have revised the Introduction (Page 5, Lines 81–83) to clarify that both the proteomic and immunocytochemical analyses focus on mucin protein cores rather than glycan structures. Specifically, LC-MS/MS analysis was performed after tryptic digestion, and the antibodies used in this study were selected to detect the protein backbone of each mucin. Glycoform-specific epitopes and glycosylation patterns were beyond the scope of the present study.

Page 5, Lines 81–83: “In this study, both proteomic and immunocytochemical analyses were designed to assess mucin protein cores rather than glycan structures.”

**Comment 3.**

*Is the selection of 8 lines (5 epithelial and 3 mesenchymal) sufficient to draw conclusions about "remodeling of mucin programs"?*

**Response:**

We appreciate the reviewer’s point. We have added the rationale for selecting eight PDAC cell lines, including five epithelial-type and three mesenchymal-type lines, to the Materials and Methods section (Page 7, Lines 123–125). We have also revised the Discussion (Page 27, Lines 464–470) to clarify that, although these lines provide internally consistent evidence of 3D-associated mucin remodeling, validation using larger panels, patient-derived organoids, and tissue specimens will be required.

Page 7, Lines 123–125: “These eight cell lines were selected to include both epithelial-classical and mesenchymal-quasi-mesenchymal phenotypes, which are commonly distinguished in transcriptomic classifications of PDAC cell lines.”

Page 27, Lines 464–470: “This study has some limitations. First, our analyses were based on a limited panel of eight established PDAC cell lines. Although this panel included both epithelial-classical and mesenchymal-quasi-mesenchymal phenotypes and provided internally consistent evidence of 3D culture-associated remodeling of mucin expression, it does not fully represent the molecular and phenotypic diversity of PDAC [10, 11, 13]. Therefore, validation using larger cell-line panels, patient-derived organoids, and clinical tissue specimens are required.”

**Comment 4.**

*Did all 8 cell lines form compact spheroids, or did some only form loose aggregates? This influences mucin expression.*

**Response:**

Spheroid morphology varied among the cell lines. We have added information regarding the day-7 morphology to the Materials and Methods section (Pages 7–8, Lines 134–138), describing whether each cell line formed compact spheroids, grape-like aggregates, or loose aggregates.

Pages 7–8, Lines 134–138: “Sphere morphology at day 7 varied among the cell lines. The epithelial PDAC cell lines (PK-1, PK-8, PK-45P, PK-59, and T3M-4) formed compact, well-circumscribed spheres, whereas KP4 and PANC-1 formed grape-like aggregates with discernible cell–cell contacts. MIA PaCa-2 cells formed loose aggregates rather than compact spheres.”

**Comment 5.**

*Was the 3D culture medium changed during these 7 days or did the spheroids grow in the same portion of medium? (section Materials and methods)*

**Response:**

We apologize for the omission. We have revised the Cell Culture subsection of the Materials and Methods (Page 7, Lines 133–134) to state explicitly that the culture medium was not changed during the 7-day 3D culture period.

Page 7, Lines 133–134: “The growth medium was not exchanged during the seven-day 3D culture period.”

**Comment 6.**

*Please standardize the tense in the Materials and Methods section. The past tense should be used, but it is mixed with the present.*

**Response:**

We have revised the Materials and Methods section (Page 6, Lines 110–116) and standardized the procedural descriptions in the past tense.

Page 6, Lines 110–116: “Fig 1. Schematic overview of the experimental design. Mucin expression profiles were identified by the proteomic analysis of eight human pancreatic ductal adenocarcinoma (PDAC) cell lines cultured under 2D and 3D conditions. Candidate mucins were validated by immunocytochemical staining using specific antibodies on cell blocks prepared from each cell line. The H-score was calculated by integrating both the proportion of positively stained cells and the intensity of immunoreactivity.”

**Comment 7.**

*Why did Authors decide to use such a high dose of trypsin?*

**Response:**

The protein-to-trypsin mass ratio used in this study was 10:1, corresponding to 2 µg trypsin for 20 µg protein, in accordance with the manufacturer’s protocol for Trypsin Platinum, Mass Spectrometry Grade (Promega, #VA9000). Since Trypsin Platinum is chemically modified to suppress autoproteolysis and is free of detectable nonspecific proteolytic activity, this condition was considered appropriate for reproducible in-solution digestion. We have added this explanation to the Proteomic Analysis subsection (Page 9, Lines 159–161).

Page 9, Lines 159–161: “Trypsin digestion was performed using Trypsin Platinum, Mass Spectrometry Grade (#VA9000; Promega), at a protein-to-trypsin mass ratio of 10:1 according to the manufacturer’s protocol.”

**Comment 8.**

*Why did Authors choose "total peptide amount" as the normalization method? Were differences in total mucin amount between 2D and 3D examined for this process?*

**Response:**

Thank you for this important comment. We have revised the Materials and Methods, Proteomic Analysis subsection (Page 11, Lines 194–195), to clarify that equal amounts of total protein were analyzed and that total-peptide-amount normalization was used to correct for technical variation in sample preparation and LC-MS/MS measurement. We have also confirmed that normalization did not substantially alter the 3D/2D mucin abundance ratios, supporting the robustness of the comparative analysis; this information has been added to the Results section (Page 15, Lines 269–274).

Page 11, Lines 194–195: “Total-peptide-amount normalization was used to correct for differences in overall peptide abundance among LC-MS/MS runs.”

Page 15, Lines 269–274: “To evaluate the effect of total-peptide-amount normalization on mucin quantification, normalized and non-normalized mucin abundance values were compared. Although normalization increased the absolute abundance values of seven mucins, excluding MUC13, by an average factor of 1.9 ± 0.3, the corresponding 3D/2D ratios were largely preserved, with an average normalized/non-normalized ratio of 1.1 ± 0.2.”

**Comment 9.**

*At what level was the FDR threshold set? Please provide this information.*

**Response:**

We have added the FDR thresholds to the Proteomic Analysis subsection (Page 10, Lines 184–186). Peptide-spectrum matches and peptides were filtered with strict and relaxed FDR thresholds of 0.01 and 0.05, respectively, and protein identification was controlled using the Protein FDR Validator with the same strict and relaxed thresholds.

Page 10, Lines 184–186: “Peptide-spectrum matches, peptides, and proteins were filtered using Percolator, Peptide Validator, and Protein FDR Validator, respectively, with strict and relaxed false discovery rate (FDR) thresholds of 0.01 and 0.05.”

**Comment 10.**

*In the Immunocytochemical Analysis section, Authors state that "heat treatment" was performed for most mucins, but details regarding the buffer used or the time it was performed are missing. Please provide this information.*

**Response:**

The Immunocytochemical Analysis subsection has been revised to specify the antigen retrieval conditions (Page 12–13, Lines 217–225). Heat-induced antigen retrieval was performed at 98°C for 40 min using BOND Epitope Retrieval Solution 1 for MUC1, MUC5AC, MUC19, and MUC20, and BOND Epitope Retrieval Solution 2 for MUC2, MUC4, and MUC13. No antigen retrieval was performed for MUC5B.

Page 12–13, Lines 217–225: “Antigen retrieval was performed by heat treatment at 98˚C for 40 min using BOND Epitope Retrieval Solution 1 (AR9961; Leica Biosystems) for MUC1, MUC5AC, MUC19, and MUC20, and BOND Epitope Retrieval Solution 2 (AR9640; Leica Biosystems) for MUC2, MUC4, and MUC13. No antigen retrieval was performed for MUC5B. Endogenous peroxidase activity was blocked by treatment with 0.3% H2O2 in water at 23˚C for 5 min. The sections were then incubated with each primary antibody for 15 min at 23˚C. Antigen detection was performed using 3,3′-diaminobenzidine tetrahydrochloride, followed by counterstaining with hematoxylin. Negative controls were generated by omitting the primary antibodies.”

**Comment 11.**

*Please provide information on the concentrations or dilutions of antibodies used.*

**Response:**

We have revised the Immunocytochemical Analysis subsection (Page 12, Lines 207–217) to include the clone, catalog number, manufacturer, and dilution or ready-to-use status of each primary antibody used for MUC1, MUC2, MUC4, MUC5AC, MUC5B, MUC13, MUC19, and MUC20.

Page 12, Lines 207–217: “The primary antibodies used for immunocytochemical staining were as follows: mouse monoclonal anti-MUC1 (Ma695; Sanbio B. V., Uden, The Netherlands; Cat# MONX10514; 1:100), mouse monoclonal anti-MUC2 (Ccp58; Leica Biosystems, Wetzlar, Germany; Cat# PA0155; ready-to-use), mouse monoclonal anti-MUC4 (8G7; Santa Cruz Biotechnology, Texas, CA, USA; Cat# sc-53945; 1:400), mouse monoclonal anti-MUC5AC (CLH2; Leica Biosystems; Cat# NCL-MUC-5AC; 1:100), mouse monoclonal anti-MUC5B (C10; Invitrogen, Waltham, MA, USA; Cat# MA5-41641; 1:100), mouse monoclonal anti-MUC13 (D-5; Santa Cruz; Cat# sc-373857; 1:200), mouse monoclonal anti-MUC19 (876013; R&D Systems, Minneapolis, MN, USA; Cat# MAB8245; 1:400), and rabbit polyclonal anti-MUC20 antibody (Invitrogen; Cat# PA5-98640; 1:500).”

**Comment 12.**

*Did Authors apply correction for multiple comparisons, e.g. Bonferroni?*

**Response:**

Thank you for pointing this out. We have reanalyzed the immunocytochemical H-score data using the Benjamini–Hochberg false-discovery-rate correction across the 64 cell-line × mucin comparisons. The Statistical analysis subsection has been revised accordingly (Page 14, Lines 243–248), and a new S1 Table has been added to show the statistical test used, raw *P* values, adjusted q values, and significance after correction. The main conclusions remain unchanged.

Page 14, Lines 243–248: “Raw P values were further adjusted across 64 cell-line × mucin comparisons using the Benjamini–Hochberg false-discovery-rate procedure, and adjusted q values < 0.05 were considered statistically significant after correction. The statistical test used for each comparison, mean H-scores, raw P values, adjusted q values, and significance after correction are provided in S1 Table.”

**Comment 13.**

*In the Results section, Authors reported that 5 mucins were detected in 2D and as many as 8 in 3D. Were these 3 additional mucins completely absent in 2D?*

**Response:**

We have revised the Results to state that MUC2, MUC5AC, and MUC13 were “not detected by LC-MS/MS” in 2D culture, rather than “absent.” This wording clarifies that these mucins may have been below the detection limit or did not meet the identification criteria under the present analytical conditions.

**Comment 14.**

*How did Authors confirm the specificity of the antibodies used to exclude cross-reactivity in the 3D model, given the high homology between MUC5AC and MUC5B?*

**Response:**

Thank you for raising this important issue. Because MUC5AC and MUC5B are highly homologous, we assessed potential antibody cross-reactivity using human tracheal tissue, in which these mucins show distinct localization patterns. The anti-MUC5AC antibody stained goblet cells of the tracheal mucosa but not submucosal glands, whereas the anti-MUC5B antibody stained submucosal glands but not mucosal goblet cells. These mutually exclusive staining patterns support the specificity of the antibodies under the conditions used in this study and suggest that the staining observed in the 3D PDAC model was unlikely to result from cross-reactivity.

**Comment 15.**

*In the PK-59 line, a decrease in MUC1 in 3D was observed, although the opposite was likely in most lines. How could Authors explain this exception in the context of MUC1 stability as a marker?*

**Response:**

We have revised the Discussion (Page 24, Lines 412–419) to clarify the interpretation of the decreased MUC1 H-score in PK-59 under 3D culture. Although the H-score decreased, MUC1 remained strongly positive in 3D-cultured PK-59 cells, indicating quantitative variation rather than loss of expression. We now describe MUC1 as a broadly maintained but quantitatively variable marker and note that its interpretation may be strengthened if combined with MUC5AC or MUC4.

Page 24, Lines 412–419: “However, MUC1 remained strongly positive in PK-59 and weakly positive in KP4 under 3D conditions, indicating that these decreases reflect quantitative variation rather than loss of expression. The reduced H-score in PK-59 may be attributable to 3D culture-associated changes in cellular organization, microenvironmental gradients, glycosylation, or epitope accessibility that influence immunocytochemical detection [19, 20, 36]. Therefore, MUC1 should be regarded as a stable but quantitatively variable marker across PDAC phenotypes and culture conditions.”

**Comment 16.**

*Authors use an H-score >5 threshold to consider a result positive. Is this a threshold chosen based on literature data for these specific antibodies, or one established by the Authors for the purposes of the study?*

**Response:**

We have revised the Statistical analysis subsection (Page 14, Lines 249–253) to clarify that the H-score >5 cutoff was defined by the authors for this study, because no universally accepted cutoff has been established for these mucin antibodies in pancreatic cancer cell lines. This threshold is approximately equivalent to weak staining in 5% of cells and is consistent with previous mucin immunohistochemical studies that used 5% positivity as a cutoff.

Page 14, Lines 249–253: “An H-score >5 was used as the threshold for positivity in the present study because no universally accepted H-score cutoff has been established for these mucin antibodies in pancreatic cancer cell lines. This value is approximately equivalent to weak staining in 5% of cells and is consistent with previous mucin immunohistochemical studies using 5% positivity as a cutoff.”

**Comment 17.**

*Authors mention the value of panel-based diagnostics. Based on these results, which specific mucin panel would be most sensitive in differentiating PDAC subtypes in 3D?*

**Response:**

Thank you for this helpful suggestion. We have revised the Discussion (Page 26, Lines 453–460) to specify that MUC1, MUC5AC, and MUC4 appear to be the most informative candidate panel in our 3D culture system. MUC1 was broadly expressed across PDAC cell lines, whereas MUC5AC and MUC4 were preferentially expressed or induced in epithelial-type lines. We therefore propose MUC1 + MUC5AC + MUC4 as a candidate panel for future validation in patient-derived organoids and tissue specimens.

## Page 26, Lines 453–460: “Based on the present H-score data, a panel combining MUC1, MUC5AC, and MUC4 may be particularly useful for differentiating PDAC phenotypes under 3D conditions. MUC1 was widely expressed across PDAC cell lines, whereas MUC5AC and MUC4 were preferentially induced or maintained in epithelial-type cell lines. In contrast, mesenchymal-type cell lines retained MUC1 expression but showed limited expression of MUC5AC and MUC4. Thus, MUC1/MUC5AC/MUC4 may represent a candidate mucin panel for future validation in patient-derived organoids and tissue-based studies.”

## Responses to Reviewer #2

**Comment.**

*The manuscript discussed Mucin expression in pancreatic ductal adenocarcinoma cell lines in 2D and 3D cultures: a proteomic and immunocytochemical analysis. The results support author theory and also sufficient techniques were performed to give insight about the manuscript data and results.*

**Response:**

We thank Reviewer #2 for the positive evaluation and for confirming that the techniques used are sufficient to support the conclusions of the study.

## Responses to Journal Requirements

**Requirement 1 — Style requirements and file naming.**

*Please ensure that your manuscript meets PLOS ONE's style requirements, including those for file naming.*

**Response:**

We have re-formatted the manuscript using the PLOS ONE main-body and title-page templates. Section ordering, heading levels, line spacing, reference formatting (numbered, in order of appearance), figure/table captions, and file naming have all been updated to match the templates.

**Requirement 2 — JSPS expansion.**

*Please expand the acronym "JSPS" so that it states the name of your funders in full.*

**Response:**

We confirm in the cover letter that JSPS stands for the Japan Society for the Promotion of Science. The full name has been used in the updated Funding Statement provided in the online submission form.

**Requirement 3 — Funding information location.**

*Please note that funding information should not appear in any section or other areas of your manuscript. We will only publish funding information present in the Funding Statement section of the online submission form. Please remove any funding-related text from the manuscript.*

**Response:**

All funding-related text has been removed from the Acknowledgements and from the manuscript. Funding details now appear only in the Funding Statement section of the online submission form.

**Requirement 4 — Supporting Information captions.**

*Please include captions for your Supporting Information files at the end of your manuscript, and update any in-text citations to match accordingly.*

**Response:**

Captions for all Supporting Information files (S1 Table and S1–S9 Figs) have been added at the end of the manuscript, immediately before the references, and all in-text citations have been verified for consistency.

**Requirement 5 — Cited works recommended by reviewers.**

*If the reviewer comments include a recommendation to cite specific previously published works, please review and evaluate these publications to determine whether they are relevant and should be cited.*

**Response:**

Reviewer #1 did not request the citation of any specific publication. No additional citations were therefore added on this basis.

**Requirement 6 — Reference list integrity.**

*Please review your reference list to ensure that it is complete and correct. If you have cited papers that have been retracted, please include the rationale for doing so in the manuscript text, or remove these references and replace them with relevant current references.*

**Response:**

We have re-checked all 49 references using PubMed, Crossref, and Retraction Watch. None of the cited articles have been retracted, and all DOIs and journal names have been verified.

We hope that the revisions, additions, and clarifications described above adequately address all points raised by the Academic Editor and the reviewers, and that the manuscript is now suitable for publication in PLOS ONE.

On behalf of all co-authors, we thank you again for the constructive review.

Sincerely,

Toshiyuki Ishiwata, M.D., Ph.D.

Corresponding Author

Tokyo Metropolitan Institute for Geriatrics and Gerontology, Tokyo, Japan
