# Supplementary material for: Mucin expression in pancreatic ductal adenocarcinoma cell lines in 2D and 3D cultures: A proteomic and immunocytochemical analysis
Source: PLoS One. 2026 Jul 16;21(7):e0353991. doi: 10.1371/journal.pone.0353991 (PMC13374910; doi:10.1371/journal.pone.0353991)
Supplement: Peer review history

# Peer review: Decision Letter 0

**DOI:** 10.1371/journal.pone.0353991.r001

**Authors:** Mahmood S Choudhery

3 May 2026

PONE-D-25-58221

Mucin expression in pancreatic ductal adenocarcinoma cell lines in 2D and 3D cultures: a proteomic and immunocytochemical analysis

PLOS One

Dear Dr. Ishiwata,

Thank you for submitting your manuscript to PLOS ONE. After careful consideration, we feel that it has merit but does not fully meet PLOS ONE’s publication criteria as it currently stands. Therefore, we invite you to submit a revised version of the manuscript that addresses the points raised during the review process.

The reviewer has raised several points requiring clarification and additional detail. Please expand the Introduction to better justify why 3D culture is expected to affect mucin expression and clarify whether analyses target protein cores, glycans, or both. Justify the use of eight cell lines and describe spheroid morphology and culture conditions, including medium changes. In Materials and Methods, ensure consistent past tense and provide details on trypsin use, normalization method, FDR threshold, antibody conditions, and multiple comparison corrections. In the Results, clarify whether additional mucins in 3D were absent in 2D, address antibody specificity, and explain the decrease of MUC1 in PK-59 cells. Finally, justify the H-score threshold and indicate which mucin panel may best distinguish PDAC subtypes in 3D models.

Please submit your revised manuscript by Jun 17 2026 11:59PM. If you will need more time than this to complete your revisions, please reply to this message or contact the journal office at plosone@plos.org. When you're ready to submit your revision, log on to https://www.editorialmanager.com/pone/ and select the 'Submissions Needing Revision' folder to locate your manuscript file.

Please include the following items when submitting your revised manuscript:

- A letter that responds to each point raised by the academic editor and reviewer(s). You should upload this letter as a separate file labeled 'Response to Reviewers'.
- A marked-up copy of your manuscript that highlights changes made to the original version. You should upload this as a separate file labeled 'Revised Manuscript with Track Changes'.
- An unmarked version of your revised paper without tracked changes. You should upload this as a separate file labeled 'Manuscript'.

If you would like to make changes to your financial disclosure, please include your updated statement in your cover letter. Guidelines for resubmitting your figure files are available below the reviewer comments at the end of this letter.

If applicable, we recommend that you deposit your laboratory protocols in protocols.io to enhance the reproducibility of your results. Protocols.io assigns your protocol its own identifier (DOI) so that it can be cited independently in the future. For instructions see: https://journals.plos.org/plosone/s/submission-guidelines#loc-laboratory-protocols. Additionally, PLOS ONE offers an option for publishing peer-reviewed Lab Protocol articles, which describe protocols hosted on protocols.io. Read more information on sharing protocols at https://plos.org/protocols?utm_medium=editorial-email&utm_source=authorletters&utm_campaign=protocols.

As the corresponding author, your ORCID iD is verified in the submission system and will appear in the published article. PLOS supports the use of ORCID, and we encourage all coauthors to register for an ORCID iD and use it as well. Please encourage your coauthors to verify their ORCID iD within the submission system before final acceptance, as unverified ORCID iDs will not appear in the published article. *Only*  the individual author can complete the verification step; PLOS staff *cannot*  verify ORCID iDs on behalf of authors.

We look forward to receiving your revised manuscript.

Kind regards,

Dr. Mahmood S Choudhery, PhD

Academic Editor

PLOS One

**Journal Requirements:**

When submitting your revision, we need you to address these additional requirements.

1. Please ensure that your manuscript meets PLOS ONE's style requirements, including those for file naming. The PLOS ONE style templates can be found at

https://journals.plos.org/plosone/s/file?id=wjVg/PLOSOne_formatting_sample_main_body.pdf and

https://journals.plos.org/plosone/s/file?id=ba62/PLOSOne_formatting_sample_title_authors_affiliations.pdf

2. Please expand the acronym “JSPS” (as indicated in your financial disclosure) so that it states the name of your funders in full.

This information should be included in your cover letter; we will change the online submission form on your behalf.

3. Please note that funding information should not appear in any section or other areas of your manuscript. We will only publish funding information present in the Funding Statement section of the online submission form. Please remove any funding-related text from the manuscript.

4. Please include captions for your Supporting Information files at the end of your manuscript, and update any in-text citations to match accordingly. Please see our Supporting Information guidelines for more information: http://journals.plos.org/plosone/s/supporting-information.

5. If the reviewer comments include a recommendation to cite specific previously published works, please review and evaluate these publications to determine whether they are relevant and should be cited. There is no requirement to cite these works unless the editor has indicated otherwise.

6. Please review your reference list to ensure that it is complete and correct. If you have cited papers that have been retracted, please include the rationale for doing so in the manuscript text, or remove these references and replace them with relevant current references. Any changes to the reference list should be mentioned in the rebuttal letter that accompanies your revised manuscript. If you need to cite a retracted article, indicate the article’s retracted status in the References list and also include a citation and full reference for the retraction notice.

Reviewers' comments:

Reviewer's Responses to Questions

**Comments to the Author**

1. Is the manuscript technically sound, and do the data support the conclusions?

The manuscript must describe a technically sound piece of scientific research with data that supports the conclusions. Experiments must have been conducted rigorously, with appropriate controls, replication, and sample sizes. The conclusions must be drawn appropriately based on the data presented.

Reviewer #1: Yes

Reviewer #2: Yes

**********

2. Has the statistical analysis been performed appropriately and rigorously?

Reviewer #1: Yes

Reviewer #2: Yes

**********

3. Have the authors made all data underlying the findings in their manuscript fully available?

The PLOS Data policy requires authors to make all data underlying the findings described in their manuscript fully available without restriction, with rare exception (please refer to the Data Availability Statement in the manuscript PDF file). The data should be provided as part of the manuscript or its supporting information, or deposited to a public repository. For example, in addition to summary statistics, the data points behind means, medians and variance measures should be available. If there are restrictions on publicly sharing data—e.g. participant privacy or use of data from a third party—those must be specified.

Reviewer #1: Yes

Reviewer #2: Yes

**********

4. Is the manuscript presented in an intelligible fashion and written in standard English?

PLOS ONE does not copyedit accepted manuscripts, so the language in submitted articles must be clear, correct, and unambiguous. Any typographical or grammatical errors should be corrected at revision, so please note any specific errors here.

Reviewer #1: Yes

Reviewer #2: Yes

**********

5. Review Comments to the Author

Please use the space provided to explain your answers to the questions above. You may also include additional comments for the author, including concerns about dual publication, research ethics, or publication ethics. (Please upload your review as an attachment if it exceeds 20,000 characters)

Reviewer #1: The manuscript presents an important issue of phenotypic plasticity in pancreatic ductal adenocarcinoma in the context of mucin expression. The presented research sheds new light on how 2D vs. 3D culture determines the proteomic profile of the tumor, which is crucial for the search for reliable biomarkers.

1. Authors state in the Introduction that 3D culture better reflects tissue architecture, but they do not explain why this would be the case for mucins. Why do Authors expect changes in mucin expression upon transition from 2D to 3D?

2. Authors state their research hypothesis regarding proteomic analysis and ICC. Will these analyses focus on the protein core or specific glycans? This is worth clarifying in the Introduction.

3. Is the selection of 8 lines (5 epithelial and 3 mesenchymal) sufficient to draw conclusions about "remodeling of mucin programs"?

4. Did all 8 cell lines form compact spheroids, or did some only form loose aggregates? This influences mucin expression.

5. Was the 3D culture medium changed during these 7 days or did the spheroids grow in the same portion of medium? (section Materials and methods)

6. Please standardize the tense in the Materials and Methods section. The past tense should be used, but it is mixed with the present.

7. Why did Authors decide to use such a high dose of trypsin?

8. Why did Authors choose "total peptide amount" as the normalization method? Were differences in total mucin amount between 2D and 3D examined for this process?

9. At what level was the FDR threshold set? Please provide this information.

10. In the Immunocytochemical Analysis section, Authors state that "heat treatment" was performed for most mucins, but details regarding the buffer used or the time it was performed are missing. Please provide this information.

11. Please provide information on the concentrations or dilutions of antibodies used.

12. Did Authors apply correction for multiple comparisons, e.g. Bonferroni?

13. In the Results section, Authors reported that 5 mucins were detected in 2D and as many as 8 in 3D. Were these 3 additional mucins completely absent in 2D?

14. How did Authors confirm the specificity of the antibodies used to exclude cross-reactivity in the 3D model, given the high homology between MUC5AC and MUC5B?

15. In the PK-59 line, a decrease in MUC1 in 3D was observed, although the opposite was likely in most lines. How could Authors explain this exception in the context of MUC1 stability as a marker?

16. Authors use an H-score >5 threshold to consider a result positive. Is this a threshold chosen based on literature data for these specific antibodies, or one established by the Authors for the purposes of the study?

17. Authors mention the value of panel-based diagnostics. Based on these results, which specific mucin panel would be most sensitive in differentiating PDAC subtypes in 3D?

Reviewer #2: The manuscript discussed Mucin expression in pancreatic ductal adenocarcinoma cell lines in 2D and 3D cultures: a proteomic and immunocytochemical analysis. The results support author theory and also sufficient techniques were performed to give insight about the manuscript data and results.

**********

6. PLOS authors have the option to publish the peer review history of their article (what does this mean?). If published, this will include your full peer review and any attached files.

If you choose “no”, your identity will remain anonymous but your review may still be made public.

**Do you want your identity to be public for this peer review?**  For information about this choice, including consent withdrawal, please see our Privacy Policy.

Reviewer #1: No

Reviewer #2: No

**********

[NOTE: If reviewer comments were submitted as an attachment file, they will be attached to this email and accessible via the submission site. Please log into your account, locate the manuscript record, and check for the action link "View Attachments". If this link does not appear, there are no attachment files.]

To ensure your figures meet our technical requirements, please review our figure guidelines: https://journals.plos.org/plosone/s/figures

You may also use PLOS’s free figure tool, NAAS, to help you prepare publication quality figures: https://journals.plos.org/plosone/s/figures#loc-tools-for-figure-preparation.

NAAS will assess whether your figures meet our technical requirements by comparing each figure against our figure specifications.

# Author comment: Author response to Decision Letter 1

**DOI:** 10.1371/journal.pone.0353991.r002

14 Jun 2026

Response to Reviewers

Manuscript ID: PONE-D-25-58221

Mucin expression in pancreatic ductal adenocarcinoma cell lines in 2D and 3D cultures: a proteomic and immunocytochemical analysis

Dear Dr. Choudhery and Reviewers,

We sincerely thank the Academic Editor and the two reviewers for their thoughtful and constructive evaluation of our manuscript. We were encouraged that both reviewers judged the work to be technically sound, statistically appropriate, with fully available data, and clearly written. We are also grateful for the reviewers’ insightful comments and suggestions, which have helped us improve the clarity, methodological transparency, and interpretation of the study.

We have addressed every point below. Reviewer comments are reproduced in italics, followed by our responses. The corresponding revisions have been incorporated into the manuscript and highlighted in the tracked-changes version. Page and line numbers refer to the revised manuscript.

Responses to Reviewer #1

We thank Reviewer #1 for their careful review of our manuscript and the seventeen specific suggestions, which have substantially strengthened the manuscript.

Comment 1.

Authors state in the Introduction that 3D culture better reflects tissue architecture, but they do not explain why this would be the case for mucins. Why do Authors expect changes in mucin expression upon transition from 2D to 3D?

Response:

Thank you for this important comment. We have revised the Introduction (Page 4, Lines 71–80) to clarify the biological rationale for comparing mucin expression between 2D and 3D cultures. Specifically, we now explain that 3D spheroids differ from 2D monolayers in cell–cell adhesion, epithelial polarity, cell density, and oxygen/nutrient gradients, all of which may influence mucin biosynthesis, glycosylation, trafficking, and secretion. We therefore present mucin remodeling as a hypothesis arising from architectural and microenvironmental differences between culture systems.

Page 4, Lines 71–80: “Mucins may be particularly susceptible to remodeling during the transition from 2D to 3D culture. This is because mucin biosynthesis depends on epithelial polarity, Golgi-resident mucin-type O-glycosylation, and polarized secretory trafficking [15-17]. These epithelial features are not fully recapitulated in flat monolayer cultures, whereas 3D spheres can partially restore tissue-like architecture, cell–cell interactions, and apical–basal polarity, while also generating gradients of oxygen and nutrients [18-20]. In addition, hypoxic and stress-responsive microenvironments in 3D cultures may contribute to the up-regulation of selected membrane-tethered and gel-forming mucins through HIF-1α-dependent pathways and endoplasmic reticulum stress or unfolded protein response signaling [21-24].”

Comment 2.

Authors state their research hypothesis regarding proteomic analysis and ICC. Will these analyses focus on the protein core or specific glycans? This is worth clarifying in the Introduction.

Response:

We have revised the Introduction (Page 5, Lines 81–83) to clarify that both the proteomic and immunocytochemical analyses focus on mucin protein cores rather than glycan structures. Specifically, LC-MS/MS analysis was performed after tryptic digestion, and the antibodies used in this study were selected to detect the protein backbone of each mucin. Glycoform-specific epitopes and glycosylation patterns were beyond the scope of the present study.

Page 5, Lines 81–83: “In this study, both proteomic and immunocytochemical analyses were designed to assess mucin protein cores rather than glycan structures.”

Comment 3.

Is the selection of 8 lines (5 epithelial and 3 mesenchymal) sufficient to draw conclusions about "remodeling of mucin programs"?

Response:

We appreciate the reviewer’s point. We have added the rationale for selecting eight PDAC cell lines, including five epithelial-type and three mesenchymal-type lines, to the Materials and Methods section (Page 7, Lines 123–125). We have also revised the Discussion (Page 27, Lines 464–470) to clarify that, although these lines provide internally consistent evidence of 3D-associated mucin remodeling, validation using larger panels, patient-derived organoids, and tissue specimens will be required.

Page 7, Lines 123–125: “These eight cell lines were selected to include both epithelial-classical and mesenchymal-quasi-mesenchymal phenotypes, which are commonly distinguished in transcriptomic classifications of PDAC cell lines.”

Page 27, Lines 464–470: “This study has some limitations. First, our analyses were based on a limited panel of eight established PDAC cell lines. Although this panel included both epithelial-classical and mesenchymal-quasi-mesenchymal phenotypes and provided internally consistent evidence of 3D culture-associated remodeling of mucin expression, it does not fully represent the molecular and phenotypic diversity of PDAC [10, 11, 13]. Therefore, validation using larger cell-line panels, patient-derived organoids, and clinical tissue specimens are required.”

Comment 4.

Did all 8 cell lines form compact spheroids, or did some only form loose aggregates? This influences mucin expression.

Response:

Spheroid morphology varied among the cell lines. We have added information regarding the day-7 morphology to the Materials and Methods section (Pages 7–8, Lines 134–138), describing whether each cell line formed compact spheroids, grape-like aggregates, or loose aggregates.

Pages 7–8, Lines 134–138: “Sphere morphology at day 7 varied among the cell lines. The epithelial PDAC cell lines (PK-1, PK-8, PK-45P, PK-59, and T3M-4) formed compact, well-circumscribed spheres, whereas KP4 and PANC-1 formed grape-like aggregates with discernible cell–cell contacts. MIA PaCa-2 cells formed loose aggregates rather than compact spheres.”

Comment 5.

Was the 3D culture medium changed during these 7 days or did the spheroids grow in the same portion of medium? (section Materials and methods)

Response:

We apologize for the omission. We have revised the Cell Culture subsection of the Materials and Methods (Page 7, Lines 133–134) to state explicitly that the culture medium was not changed during the 7-day 3D culture period.

Page 7, Lines 133–134: “The growth medium was not exchanged during the seven-day 3D culture period.”

Comment 6.

Please standardize the tense in the Materials and Methods section. The past tense should be used, but it is mixed with the present.

Response:

We have revised the Materials and Methods section (Page 6, Lines 110–116) and standardized the procedural descriptions in the past tense.

Page 6, Lines 110–116: “Fig 1. Schematic overview of the experimental design. Mucin expression profiles were identified by the proteomic analysis of eight human pancreatic ductal adenocarcinoma (PDAC) cell lines cultured under 2D and 3D conditions. Candidate mucins were validated by immunocytochemical staining using specific antibodies on cell blocks prepared from each cell line. The H-score was calculated by integrating both the proportion of positively stained cells and the intensity of immunoreactivity.”

Comment 7.

Why did Authors decide to use such a high dose of trypsin?

Response:

The protein-to-trypsin mass ratio used in this study was 10:1, corresponding to 2 µg trypsin for 20 µg protein, in accordance with the manufacturer’s protocol for Trypsin Platinum, Mass Spectrometry Grade (Promega, #VA9000). Since Trypsin Platinum is chemically modified to suppress autoproteolysis and is free of detectable nonspecific proteolytic activity, this condition was considered appropriate for reproducible in-solution digestion. We have added this explanation to the Proteomic Analysis subsection (Page 9, Lines 159–161).

Page 9, Lines 159–161: “Trypsin digestion was performed using Trypsin Platinum, Mass Spectrometry Grade (#VA9000; Promega), at a protein-to-trypsin mass ratio of 10:1 according to the manufacturer’s protocol.”

Comment 8.

Why did Authors choose "total peptide amount" as the normalization method? Were differences in total mucin amount between 2D and 3D examined for this process?

Response:

Thank you for this important comment. We have revised the Materials and Methods, Proteomic Analysis subsection (Page 11, Lines 194–195), to clarify that equal amounts of total protein were analyzed and that total-peptide-amount normalization was used to correct for technical variation in sample preparation and LC-MS/MS measurement. We have also confirmed that normalization did not substantially alter the 3D/2D mucin abundance ratios, supporting the robustness of the comparative analysis; this information has been added to the Results section (Page 15, Lines 269–274).

Page 11, Lines 194–195: “Total-peptide-amount normalization was used to correct for differences in overall peptide abundance among LC-MS/MS runs.”

Page 15, Lines 269–274: “To evaluate the effect of total-peptide-amount normalization on mucin quantification, normalized and non-normalized mucin abundance values were compared. Although normalization increased the absolute abundance values of seven mucins, excluding MUC13, by an average factor of 1.9 ± 0.3, the corresponding 3D/2D ratios were largely preserved, with an average normalized/non-normalized ratio of 1.1 ± 0.2.”

Comment 9.

At what level was the FDR threshold set? Please provide this information.

Response:

We have added the FDR thresholds to the Proteomic Analysis subsection (Page 10, Lines 184–186). Peptide-spectrum matches and peptides were filtered with strict and relaxed FDR thresholds of 0.01 and 0.05, respectively, and protein identification was controlled using the Protein FDR Validator with the same strict and relaxed thresholds.

Page 10, Lines 184–186: “Peptide-spectrum matches, peptides, and proteins were filtered using Percolator, Peptide Validator, and Protein FDR Validator, respectively, with strict and relaxed false discovery rate (FDR) thresholds of 0.01 and 0.05.”

Comment 10.

In the Immunocytochemical Analysis section, Authors state that "heat treatment" was performed for most mucins, but details regarding the buffer used or the time it was performed are missing. Please provide this information.

Response:

The Immunocytochemical Analysis subsection has been revised to specify the antigen retrieval conditions (Page 12–13, Lines 217–225). Heat-induced antigen retrieval was performed at 98°C for 40 min using BOND Epitope Retrieval Solution 1 for MUC1, MUC5AC, MUC19, and MUC20, and BOND Epitope Retrieval Solution 2 for MUC2, MUC4, and MUC13. No antigen retrieval was performed for MUC5B.

Page 12–13, Lines 217–225: “Antigen retrieval was performed by heat treatment at 98˚C for 40 min using BOND Epitope Retrieval Solution 1 (AR9961; Leica Biosystems) for MUC1, MUC5AC, MUC19, and MUC20, and BOND Epitope Retrieval Solution 2 (AR9640; Leica Biosystems) for MUC2, MUC4, and MUC13. No antigen retrieval was performed for MUC5B. Endogenous peroxidase activity was blocked by treatment with 0.3% H2O2 in water at 23˚C for 5 min. The sections were then incubated with each primary antibody for 15 min at 23˚C. Antigen detection was performed using 3,3′-diaminobenzidine tetrahydrochloride, followed by counterstaining with hematoxylin. Negative controls were generated by omitting the primary antibodies.”

Comment 11.

Please provide information on the concentrations or dilutions of antibodies used.

Response:

We have revised the Immunocytochemical Analysis subsection (Page 12, Lines 207–217) to include the clone, catalog number, manufacturer, and dilution or ready-to-use status of each primary antibody used for MUC1, MUC2, MUC4, MUC5AC, MUC5B, MUC13, MUC19, and MUC20.

Page 12, Lines 207–217: “The primary antibodies used for immunocytochemical staining were as follows: mouse monoclonal anti-MUC1 (Ma695; Sanbio B. V., Uden, The Netherlands; Cat# MONX10514; 1:100), mouse monoclonal anti-MUC2 (Ccp58; Leica Biosystems, Wetzlar, Germany; Cat# PA0155; ready-to-use), mouse monoclonal anti-MUC4 (8G7; Santa Cruz Biotechnology, Texas, CA, USA; Cat# sc-53945; 1:400), mouse monoclonal anti-MUC5AC (CLH2; Leica Biosystems; Cat# NCL-MUC-5AC; 1:100), mouse monoclonal anti-MUC5B (C10; Invitrogen, Waltham, MA, USA; Cat# MA5-41641; 1:100), mouse monoclonal anti-MUC13 (D-5; Santa Cruz; Cat# sc-373857; 1:200), mouse monoclonal anti-MUC19 (876013; R&D Systems, Minneapolis, MN, USA; Cat# MAB8245; 1:400), and rabbit polyclonal anti-MUC20 antibody (Invitrogen; Cat# PA5-98640; 1:500).”

Comment 12.

Did Authors apply correction for multiple comparisons, e.g. Bonferroni?

Response:

Thank you for pointing this out. We have reanalyzed the immunocytochemical H-score data using the Benjamini–Hochberg false-discovery-rate correction across the 64 cell-line × mucin comparisons. The Statistical analysis subsection has been revised accordingly (Page 14, Lines 243–248), and a new S1 Table has been added to show the statistical test used, raw P values, adjusted q values, and significance after correction. The main conclusions remain unchanged.

Page 14, Lines 243–248: “Raw P values were further adjusted across 64 cell-line × mucin comparisons using the Benjamini–Hochberg false-discovery-rate procedure, and adjusted q values < 0.05 were considered statistically significant after correction. The statistical test used for each comparison, mean H-scores, raw P values, adjusted q values, and significance after correction are provided in S1 Table.”

Comment 13.

In the Results section, Authors reported that 5 mucins were detected in 2D and as many as 8 in 3D. Were these 3 additional mucins completely absent in 2D?

Response:

We have revised the Results to state that MUC2, MUC5AC, and MUC13 were “not detected by LC-MS/MS” in 2D culture, rather than “absent.” This wording clarifies that these mucins may have been below the detection limit or did not meet the identification criteria under the present analytical conditions.

Comment 14.

How did Authors confirm the specificity of the antibodies used to exclude cross-reactivity in the 3D model, given the high homology between MUC5AC and MUC5B?

Response:

Thank you for raising this important issue. Because MUC5AC and MUC5B are highly homologous, we assessed potential antibody cross-reactivity using human tracheal tissue, in which these mucins show distinct localization patterns. The anti-MUC5AC antibody stained goblet cells of the tracheal mucosa but not submucosal glands, whereas the anti-MUC5B antibody stained submucosal glands but not mucosal goblet cells. These mutually exclusive staining patterns support the specificity of the antibodies under the conditions used in this study and suggest that the staining observed in the 3D PDAC model was unlikely to result from cross-reactivity.

Comment 15.

In the PK-59 line, a decrease in MUC1 in 3D was observed, although the opposite was likely in most lines. How could Authors explain this exception in the context of MUC1 stability as a marker?

Response:

We have revised the Discussion (Page 24, Lines 412–419) to clarify the interpretation of the decreased MUC1 H-score in PK-59 under 3D culture. Although the H-score decreased, MUC1 remained strongly positive in 3D-cultured PK-59 cells, indicating quantitative variation rather than loss of expression. We now describe MUC1 as a broadly maintained but quantitatively variable marker and note that its interpretation may be strengthened if combined with MUC5AC or MUC4.

Page 24, Lines 412–419: “However, MUC1 remained strongly positive in PK-59 and weakly positive in KP4 under 3D conditions, indicating that these decreases reflect quantitative variation rather than loss of expression. The reduced H-score in PK-59 may be attributable to 3D culture-associated changes in cellular organization, microenvironmental gradients, glycosylation, or epitope accessibility that influence immunocytochemical detection [19, 20, 36]. Therefore, MUC1 should be regarded as a stable but quantitatively variable marker across PDAC phenotypes and culture conditions.”

Comment 16.

Authors use an H-score >5 threshold to consider a result positive. Is this a threshold chosen based on literature data for these specific antibodies, or one established by the Authors for the purposes of the study?

Response:

We have revised the Statistical analysis su

Attachment Submitted filename: 6-15-2026__Response_to_Reviewers_for submission.docx

# Editor report: Decision Letter 1

**DOI:** 10.1371/journal.pone.0353991.r003

**Authors:** Mahmood S Choudhery, Mahmood S Choudhery

1 Jul 2026

Mucin expression in pancreatic ductal adenocarcinoma cell lines in 2D and 3D cultures: a proteomic and immunocytochemical analysis

PONE-D-25-58221R1

Dear Dr. Ishiwata,

We’re pleased to inform you that your manuscript has been judged scientifically suitable for publication and will be formally accepted for publication once it meets all outstanding technical requirements.

Within one week, you’ll receive an e-mail detailing the required amendments. When these have been addressed, you’ll receive a formal acceptance letter and your manuscript will be scheduled for publication.

An invoice will be generated when your article is formally accepted. Please note, if your institution has a publishing partnership with PLOS and your article meets the relevant criteria, all or part of your publication costs will be covered. Please make sure your user information is up-to-date by logging into Editorial Manager at Editorial Manager® and clicking the ‘Update My Information' link at the top of the page. For questions related to billing, please contact billing support.

If your institution or institutions have a press office, please notify them about your upcoming paper to help maximize its impact. If they’ll be preparing press materials, please inform our press team as soon as possible -- no later than 48 hours after receiving the formal acceptance. Your manuscript will remain under strict press embargo until 2 pm Eastern Time on the date of publication. For more information, please contact onepress@plos.org.

Kind regards,

Mahmood S Choudhery, PhD

Academic Editor

PLOS One

Additional Editor Comments (optional):

Reviewers' comments:

# Editor report: Acceptance letter

**DOI:** 10.1371/journal.pone.0353991.r004

**Authors:** Mahmood S Choudhery, Mahmood S Choudhery

PONE-D-25-58221R1

PLOS One

Dear Dr. Ishiwata,

I'm pleased to inform you that your manuscript has been deemed suitable for publication in PLOS One. Congratulations! Your manuscript is now being handed over to our production team.

At this stage, our production department will prepare your paper for publication. This includes ensuring the following:

* All references, tables, and figures are properly cited

* All relevant supporting information is included in the manuscript submission,

* There are no issues that prevent the paper from being properly typeset

You will receive further instructions from the production team, including instructions on how to review your proof when it is ready. Please keep in mind that we are working through a large volume of accepted articles, so please give us a few days to review your paper and let you know the next and final steps.

Lastly, if your institution or institutions have a press office, please let them know about your upcoming paper now to help maximize its impact. If they'll be preparing press materials, please inform our press team within the next 48 hours. Your manuscript will remain under strict press embargo until 2 pm Eastern Time on the date of publication. For more information, please contact onepress@plos.org.

You will receive an invoice from PLOS for your publication fee after your manuscript has reached the completed accept phase. If you receive an email requesting payment before acceptance or for any other service, this may be a phishing scheme. Learn how to identify phishing emails and protect your accounts at https://explore.plos.org/phishing.

If we can help with anything else, please email us at customercare@plos.org.

Thank you for submitting your work to PLOS ONE and supporting open access.

Kind regards,

PLOS ONE Editorial Office Staff

on behalf of

Dr. Mahmood S Choudhery

Academic Editor

PLOS One
